# 808 nm-activable core@multishell upconverting nanoparticles with enhanced stability for efficient photodynamic therapy

**DOI:** 10.1186/s12951-020-00640-3

**Published:** 2020-06-05

**Authors:** Raquel Martínez, Ester Polo, Silvia Barbosa, Pablo Taboada, Pablo del Pino, Beatriz Pelaz

**Affiliations:** 1 Centro Singular de Investigación en Química Biolóxica e Materiais Moleculares (CiQUS), Universidade de Santiago de Compostela, 15782 Santiago, Spain; 2 Grupo de Física de Coloides y Polímeros, Departamento de Física de Partículas, Universidade de Santiago de Compostela, 15782 Santiago, Spain; 3 Instituto de Investigaciones Sanitarias, Universidade de Santiago de Compostela, 15782 Santiago, Spain; 4 Grupo de Física de Coloides y Polímeros, Departamento de Inorgánica, Universidade de Santiago de Compostela, 15782 Santiago, Spain

**Keywords:** Upconverting nanoparticles, Photodynamic therapy, Photosensitizers, Click chemistry, Polymer coating

## Abstract

**Background:**

The unique upconversion properties of rare-earth-doped nanoparticles offers exciting opportunities for biomedical applications, in which near-IR remote activation of biological processes is desired, including in vivo bioimaging, optogenetics, and light-based therapies. Tuning of upconversion in purposely designed core–shell nanoparticles gives access to biological windows in biological tissue. In recent years there have been several reports on NIR-excitable upconverting nanoparticles capable of working in biological mixtures and cellular settings. Unfortunately, most of these nanosystems are based on ytterbium’s upconversion at 980 nm, concurrent with water’s absorption within the first biological window. Thus, methods to produce robust upconverting nanoplatforms that can be efficiently excited with other than 980 nm NIR sources, such as 808 nm and 1064 nm, are required for biomedical applications.

**Results:**

Herein, we report a synthetic method to produce aqueous stable upconverting nanoparticles that can be activated with 808 nm excitation sources, thus avoiding unwanted heating processes due to water absorbance at 980 nm. Importantly, these nanoparticles, once transferred to an aqueous environment using an amphiphilic polymer, remain colloidally stable for long periods of time in relevant biological media, while keeping their photoluminescence properties. The selected polymer was covalently modified by click chemistry with two FDA-approved photosensitizers (Rose Bengal and Chlorin e6), which can be efficiently and simultaneously excited by the light emission of our upconverting nanoparticles. Thus, our polymer-functionalization strategy allows producing an 808 nm-activable photodynamic nanoplatform. These upconverting nanocomposites are preferentially stored in acidic lysosomal compartments, which does not negatively affect their performance as photodynamic agents. Upon 808 nm excitation, the production of reactive oxidative species (ROS) and their effect in mitochondrial integrity were demonstrated.

**Conclusions:**

In summary, we have demonstrated the feasibility of using photosensitizer-polymer-modified upconverting nanoplatforms that can be activated by 808 nm light excitation sources for application in photodynamic therapy. Our nanoplatforms remain photoactive after internalization by living cells, allowing for 808 nm-activated ROS generation. The versatility of our polymer-stabilization strategy promises a straightforward access to other derivatizations (for instance, by integrating other photosensitizers or homing ligands), which could synergistically operate as multifunctional photodynamic platforms nanoreactors for in vivo applications.

## Background

The potential of rare-earth (RE) upconverting nanoparticles (UCNPs) as next generation luminophores is unique [1]. This potential relies on their distinctive optical properties, including the absence of blinking, low excitation rate compared with organic dyes or semiconductor nanocrystals [2], excitability by wavelengths within biological windows of tissue penetration, and high signal-to-noise ratio, among others [3]. However, some challenges related to their translatability for diagnosis and therapy remain troublesome, such as the NIR wavelength required for upconversion activation. Many approaches have been recently developed to shift this NIR excitation wavelength from the commonly used 980 nm to 808 nm [4–6], where the water absorbance is ca. 5% compared with the absorbance at 980 nm [7], avoiding thereby undesired heating processes and losses of the excitation efficiency due to water absorption. One of the most successful solutions relies on the inclusion of Nd^3+^ ions as sensitizers, taking advantage of their 800 nm characteristic excitation band. It has been demonstrated that Nd^3+^ is able to transfer energy successfully to Yb^3+^ and this one to Er^3+^ [8]. However, the limitation of this approach is the presence of deleterious energy transfer processes that happen when all the lanthanide ions sit at the same matrix. This issue has been overcome by the introduction of core@shell structures that allow for the use of high doping percentages [9, 10].

Another open challenge is related to UCNP stability and upconversion efficiency in water and biologically relevant media [11, 12]. In this direction, manifold surface coatings have been explored in order to not only properly stabilize the UCNPs in these media, but to preserve their luminescent properties [4, 11, 13–16]. Notice that the synthesis of high-quality colloidal UCNPs is typically carried out in organic solvents with relatively high boiling points (e.g., mixtures of 1-octadecene and oleic acid) using thermal decomposition methods [12]. These synthetic processes yield UCNPs capped with aliphatic ligands as, for example, oleic acid, which provide colloidal stability in organic solvents; it is thus mandatory to carry out a water transfer process if the UCNPs are to be used in biological fluids. To perform this step, two main strategies can be used: (i) a ligand exchange process consisting in the replacement of the hydrophobic ligands attached on the NP surface; [17] and (ii) the growth of a hydrophilic outer shell on top of the primary hydrophobic capping ligands [18]. The first approach deals with modifications directly performed onto the UCNP’s surface, in which the generation of defects and efficiency losses are more likely to happen compared with the second approach. One of the most versatile strategies for the UCNPs’ stabilization, as well as many other inorganic NPs, entails the use of amphiphilic polymers such as poly(isobutylene-alt-maleic anhydride) dodecyl grafted (PMA from now) [3, 19].

As mentioned above, the potential of UCNPs as therapeutic agents is promising, particularly, as photodynamic (PDT) agents. Moreover, their use for other purposes such as optogenetics has been also evaluated [20]. Nevertheless, many of the literature precedents involved the use of 980 nm-excitable UCNPs [21–23]. The use of photosensitizers (PS) covalently linked to UCNPs allows for the design of more efficient multifunctional PDT agents, which combined with 808 nm-photoactivable UCNPs provides for new opportunities to develop more effective, translational therapeutic approaches [24–27]. The use of 808 nm-gated PDT agents based on UCNPs has been previously explored by using both PS-filled porous shells [28, 29], and PS-modified polymer-based coatings [10, 26, 30–32]. We chose to modify our PMA with dibenzocyclooctyne (DBCO) groups, thereby providing for a versatile platform to attach azide-containing molecules by copper-free click chemistry.

**Scheme 1:**
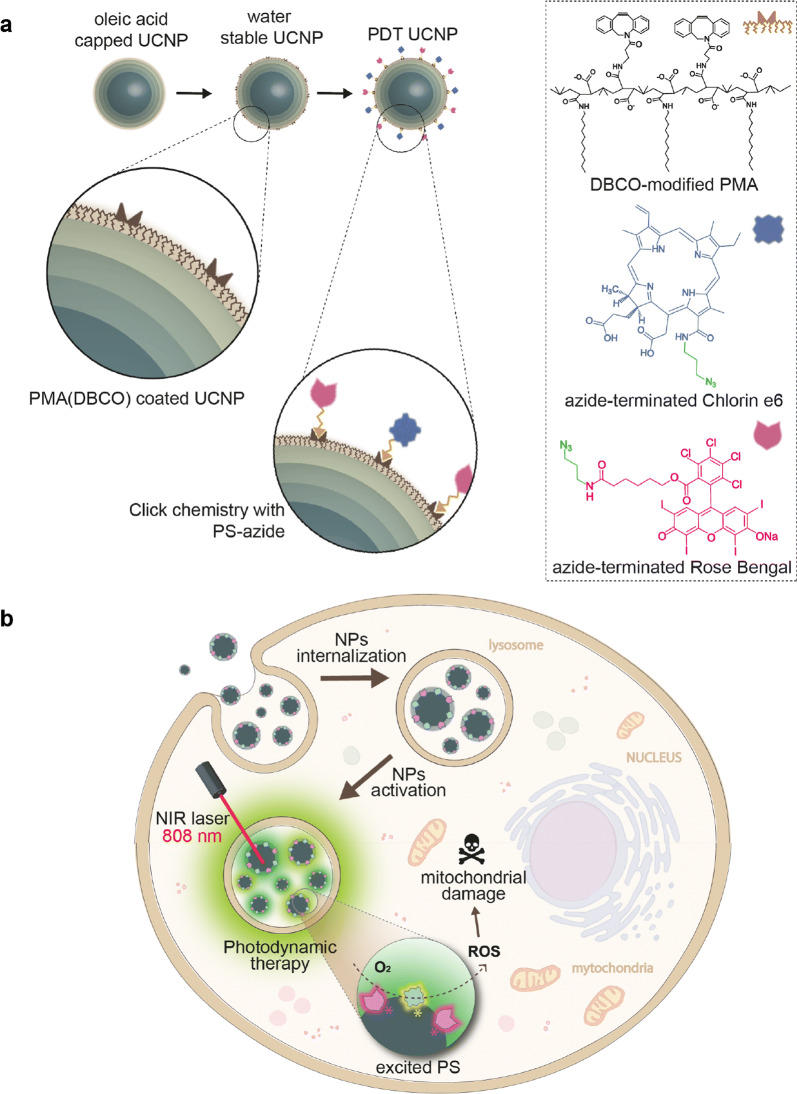
**a** Illustration of the synthetic steps to produce UCNPs, their water transfer, and functionalization with two PSs (Rose Bengal and Chlorin e6). **b** Intracellular location of UCNPs in endocytic compartments and PDT therapy induced by 808 nm laser irradiation, leading to the impairment of mitochondria

In the present work, the synthesis of 808 nm-activable UCNPs using a layer-by-layer methodology, without the use of trifluoroacetate precursors, is proposed. These NPs were water-transferred using a polymer coating strategy based on the amphiphilic DBCO-modified polymer PMA. Taking advantage of the PMA’s functional flexibility, two different FDA-approved photosensitizers, Rose Bengal and Chlorin e6, were covalently attached to the UCNPs by click chemistry, to produce PDT nanoprobes for 808 nm-gated intracellular generation of reactive oxidative species (ROS) with spatiotemporal resolution (Scheme 1).

## Results and discussion

**Fig. 1:**
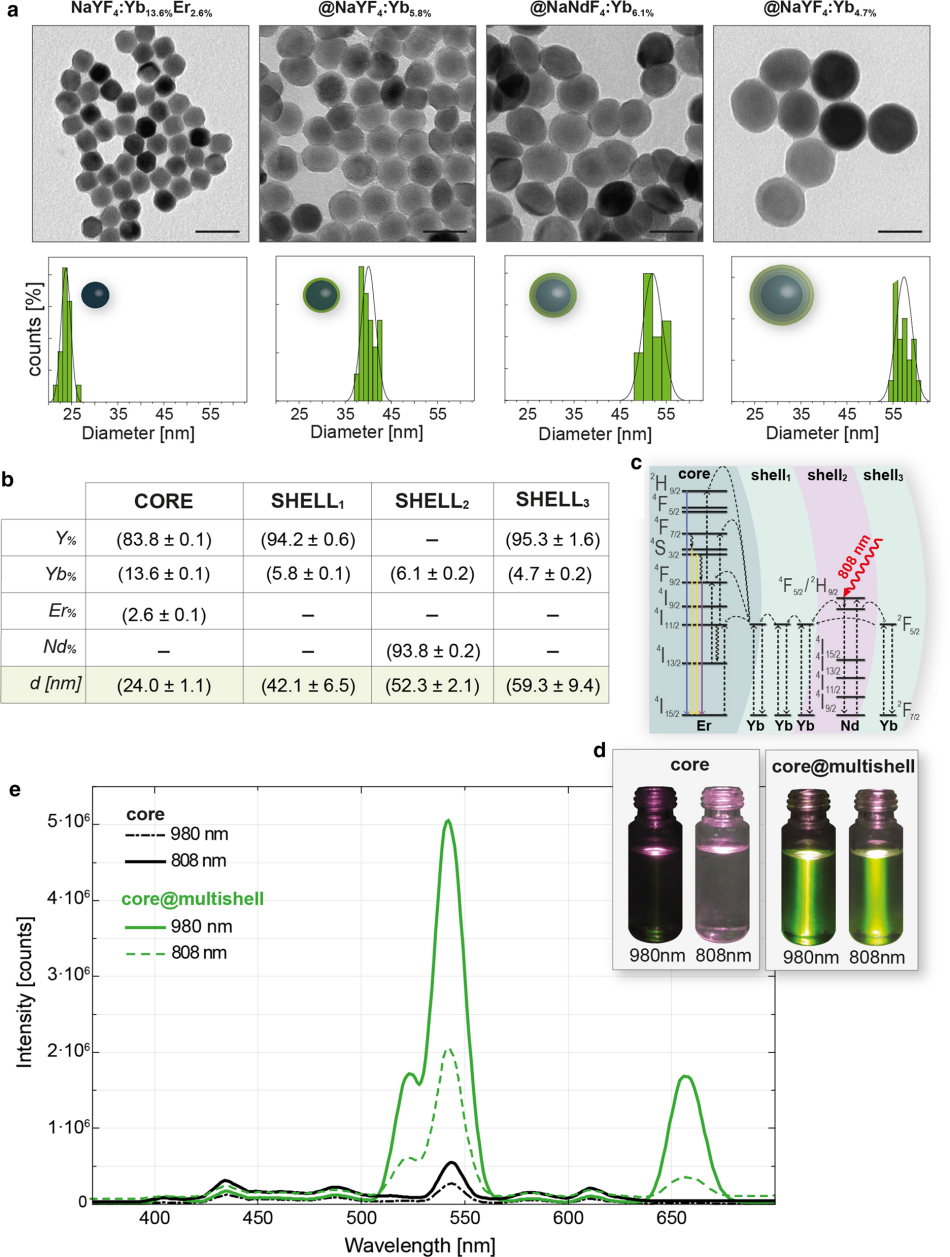
**a** Transmission EM (TEM) micrographs of the layer by layer process from cores (left) to the multishell particles (right). Scale bars correspond to 50 nm. The corresponding histograms for the synthetic steps are depicted in green bars below each image. **b** Table reflecting UCNPs composition, including the diameter or thickness of the core or the layer based on TEM images, and the individual core and shell composition as determined by ICP-MS. **c** Schematic representation of the upconversion mechanism of the multi-shell nanoplatforms. **d** Photographs of solutions of cores (left) and core@multishell UCNPs (right) under with a 980 nm- and 808 nm-excitation lasers, respectively. **e** Emission spectra of cores (black lines) and core@multishell UCNPs (green lines) in chloroform (~ 5 nM particles) under 808 nm and 980 nm excitation (1 W cm^−2^), respectively

Lanthanide-doped core@multishell UCNPs with inorganic diameter ~ 60 nm were synthesized in organic solvents, aiming to specifically obtain NaYF_4_:Yb_18%_Er_2%_ @NaYF_4_:Yb_10%_ @NaNdF_4_:Yb_10%_ @NaYF_4_:Yb_10%_ lumiprobes, featuring a highly effective 808 nm to visible conversion, as previously reported; [10] such structure entails (see Additional file 1: Table S1): (i) an upconversion luminescence core; (ii) an energy transfer layer (shell_1_); (iii) an energy absorption layer (shell_2_); (iv) an energy transfer and luminescence reduction layer (shell_3_). We followed the thermal decomposition route (see experimental setup in Additional file 1: Figure S1), adapting previously reported layer-by-layer protocols [33], in which the commonly used trifluoroacetate precursors were replaced by chloride precursors. The replacement of trifluoroacetate reactants avoids the potential decomposition of the precursors into HF gas or fluorinated species during the reaction at high temperature [34]. Firstly, nanocores with diameter ~ 24 nm were produced (Additional file 1: Figures S2 and S3), and their X-ray diffraction pattern confirmed the expected hexagonal crystalline structure (see Additional file 1: Figure S4 and Table S2) [35]. The shell growth was characterized by electron microscopy (EM, both in transmission and scanning modes, see Fig. 1a, and Additional file 1: Figure S2). We combined EM data and compositional analysis by inductively coupled plasma mass spectrometry (ICP-MS), to determine the composition of each shell and the core (Additional file 1: Tables S3–S7). The diameters and the individual shell compositions are summarized in Fig. 1b (for additional details see Additional file 1: Tables S6 and S7). The obtained composition corresponds to NaYF_4_:Yb_13.6%_Er_2.6%_ @NaYF_4_:Yb_5.8%_ @NaNdF_4_:Yb_6.1%_ @NaYF_4_:Yb_4.7%_. Based on this, and the size previously determined, the UCNPs’ molecular weight was estimated (MW ~ 30·10^7^ g mol^−1^), allowing for an accurate determination of the UCNPs’ concentration. The MW value experimentally obtained (see Additional file 1: Table S8) here is in good agreement with the theoretical value recently calculated by Mackenzie et al. [36], which have developed a theoretical method to determine the UCNPs’ MW. They described that UCNPs (NaYF_4_:RE) with diameters between 10 to 45 nm present MWs ranging from 10^6^ to 10^8^ g mol^−1^. The experimental determination of NP’s MW is critical for their biomedical application [37]. In our case, it provides for control over the dose of UCNPs used in the experiments involving living cells.

As expected, our nanocores (NaYF_4_:Yb_13.6%_Er_2.6%_) were excitable at 980 nm but showed residual emission when they were excited with an 808 nm laser (see photoemission mechanism illustrated in Fig. 1c, previously discussed by Lin et al. [10]), as it can be qualitatively observed in photographs of cores solutions under the two light sources (Fig. 1d), as well as in the corresponding emission spectra (Fig. 1e). On the other hand, the final core@multishell UCNPs were activable under the excitation of both wavelengths (Fig. 1d, e). The emission spectra of the core@multishell UCNPs showed that the emission peaks and intensity ratios remained unaltered for both laser sources (using ~ 5 nM UCNP solution and an irradiance of 1 W cm^−2^). In all these cases, the most intense peaks were centered at 522, 542 and 657 nm. These values and intensities are in good agreement with reported samples with a matrix of NaYF_4_ doped with Er^3+^ as activator and Yb^3+^ as sensitizer [34].

The synthesized UCNPs were hydrophobically capped (oleic acid); therefore, a step to water-transfer them was required. The stabilization of UCNPs in relevant biological media while keeping their photoluminescence properties is still an open challenge [12]. Manifold strategies have been explored including ligand exchange processes [11, 38], and the use of amphiphilic polymers [3, 16]. Considering this, the use of an amphiphilic polymer (PMA) was selected. The polymer coating technique has been employed to successfully water transfer most types of inorganic nanoparticles [18]. This approach has been recently proven to be also valid for the water transference of NPs with diameter larger than 20 nm [39], and extremely water-sensitive photoluminescence NPs such halide nanoperovskites [40]. The selection of this polymer was based on the following points: (i) this technique has been widely used to stabilize quantum dots, minimally affecting their optoelectronic properties, and recently, for the stabilization of 980-activable UCNPs; [3] (ii) high yield of NPs with robust colloidal stability, even in complex biological media, can be obtained; [41] and (iii) offers a very versatile platform to perform the covalent bioconjugation of different ligands of interest [42]. In order transfer them to water, we used the amphiphilic polymer PMA in which the polymer-grafter hydrophobic chains (dodecylamine) are entangled with the oleic acid chains of the as-synthesized UCNPs for colloidal stabilization. This phenomenon is explained in detail in SI. The PMA used here was modified with DBCO pendant groups before the coating of the UCNPs (see “Materials and methods”; Additional file 1: Figures S5 and S6). Thus, the resulted polymer-coated NPs could be easily modified with azide-containing ligands using a copper-free click chemistry reaction.

**Fig. 2:**
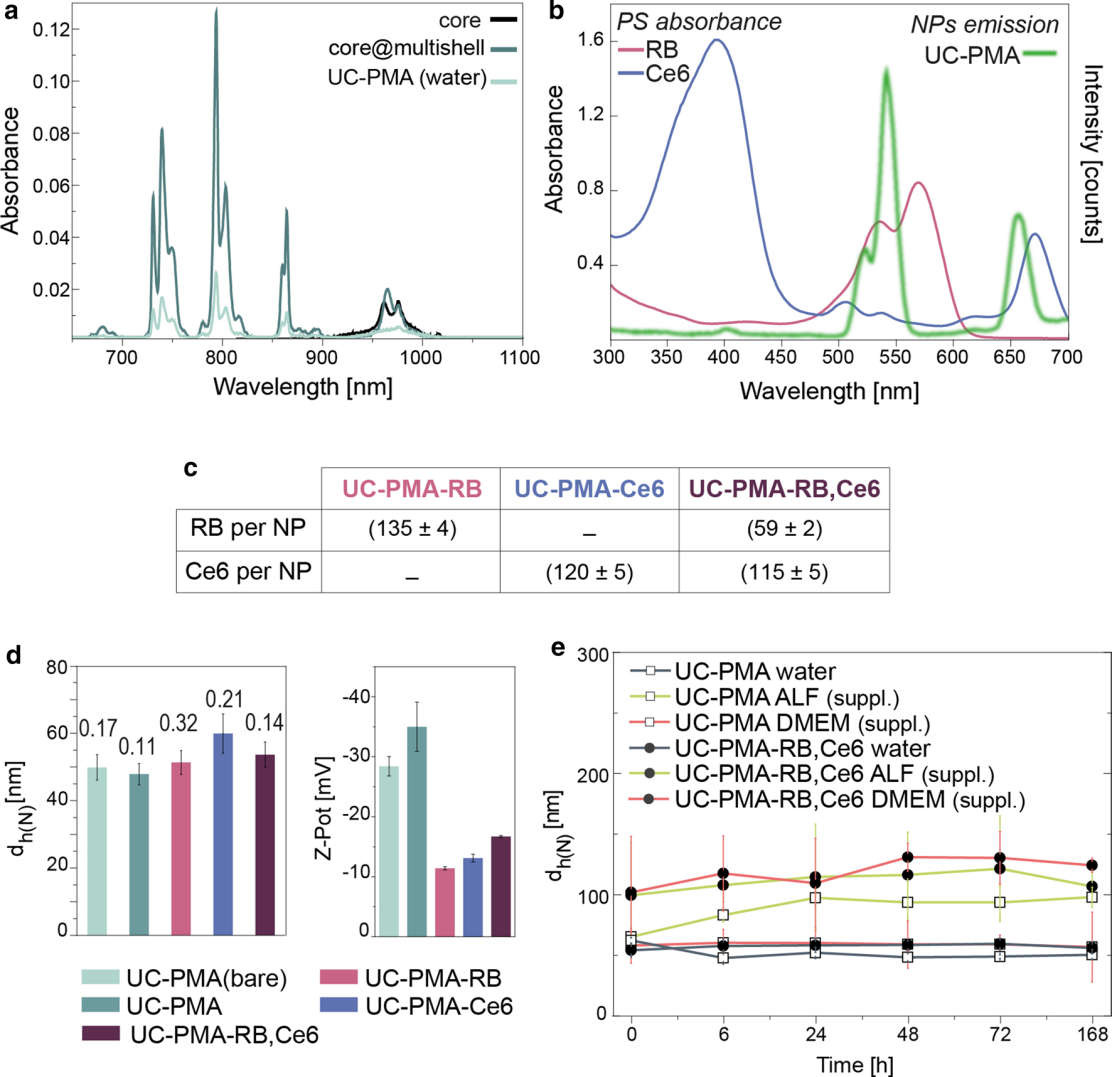
**a** Absorbance of core, core@multishell, and UC-PMA NPs in chloroform and water. **b** Overlay of the Rose Bengal and Chlorin e6 absorbance and UC-PMA emission in water. **c** Summary of the PSs attached per UCNP. **d** DLS and LDA values of the different ligands’ combinations attached to the UCNPs; each bar is labeled with the corresponding polydispersity index (PDI). **e** Hydrodynamic diameter values (number-weighted) overtime in different media (water, blue; supplemented ALF, green; supplemented DMEM, orange) of UC-PMA (white dots) and UC-PMA-RB,Ce6 (black dots) (raw data showed in Additional file 1: Table S9)

After the PMA-coating of the UCNPs (UC-PMA), the absorbance and the emission spectra were recorded (Fig. 2a). For a solution with the same concentration, the absorbance after the water transference dropped to ~ 25% of the initial absorbance of uncoated NPs in chloroform. Interestingly, the relative peak intensity, the ratio of the peaks, remains unaltered after the coating, in agreement with other water-transferred UCNPs [38]. Emission peaks position remained unaltered after the water transfer, but as it happens with the absorbance, the emission rate decreased (see Additional file 1: Figure S7).

To maximize the efficiency of the PDT potential of the UCNPs, two PSs, Rose Bengal (RB from now) and Chlorin e6 (Ce6 from now) were selected as they match the most intense emission peaks of the UCNPs upon NIR excitation (see Fig. 2b and Additional file 1). Both PSs were modified to include an azide group in their structure (Additional file 1: Figures S5 and S6) for their covalent binding to the PMA. Three different probes containing one or both PS were prepared: UC-RB, UC-Ce6 and UC-RB,Ce6 (see Additional file 1: Figure S8). During the conjugation process and due to its high efficiency compared with other bioconjugation techniques [42], it is important to have a good control over the following parameters: (i) NP:PS ratios above 1:750 will decrease dramatically the hydrophilicity of the NPs, thereby impairing their colloidal stability and leading to the partial aggregation of the sample; [42] (ii) the final NP concentration during the reaction (not higher than 16 nM); and (iii) to keep the H_2_O:DMSO ratio during the reaction below 4:1. A quantitative determination of the number of PS attached in each probe was determined, using appropriate calibration curves (Additional file 1: Figure S9). When modifying the NPs with one PS type (RB or Ce6) approximately 100 molecules were linked per NPs. This number is in good agreement with the size and the loading efficiency compared with other modified-PMA coated NPs [42, 43]. Interestingly, when both PS were used to modify the UCNPs, the efficiency for the Ce6 binding remained constant, ~ 100 Ce6 NP^−1^, but the number of RB molecules per NP dropped to 50 (see Fig. 2c). This might indicate some priority in Ce6 binding over RB when there is a competition during the bioconjugation that is not present when the UCNPs are modified just with one PS type.

After the PS’s conjugation, colloidal characterization using SEM (Additional file 1: Figure S10), dynamic light scattering (DLS) and laser Doppler anemometry (LDA) were performed in water. Changes in the hydrodynamic diameter (d_h_) were not significant, but in the functionalized NPs, a decrease in the ζ-potential value was observed in all the cases from ~ − 30 to − 15 mV (see Fig. 2d, Additional file 1: Figure S11 and Table S9 for the obtained values), consistent with PS attachment.

Finally, the colloidal stability of the UC-PMA and UC-PMA-RB,Ce6 over time, for at least 1 week, was studied. To do so, four different aqueous-based media were selected; that is, water, Dulbecco’s modified Eagle’s medium (DMEM) supplemented with fetal bovine serum (FBS, 10%), and artificial lysosomal fluid (ALF) with and without FBS (10%) (see Fig. 2e). Both types of NPs remained stable over the incubation time in water. However, as reported previously, the PMA-coated NPs were unstable in medium without FBS (see Additional file 1: Figure S12) [39]. Both samples remained stable in the complete media, DMEM and ALF. As expected, an increase in size was observed in both cases likely due to the unspecific adsorption of proteins [44], but these size variation remained constant along the observation time, confirming the nanoplatform’s stability.

**Fig. 3:**
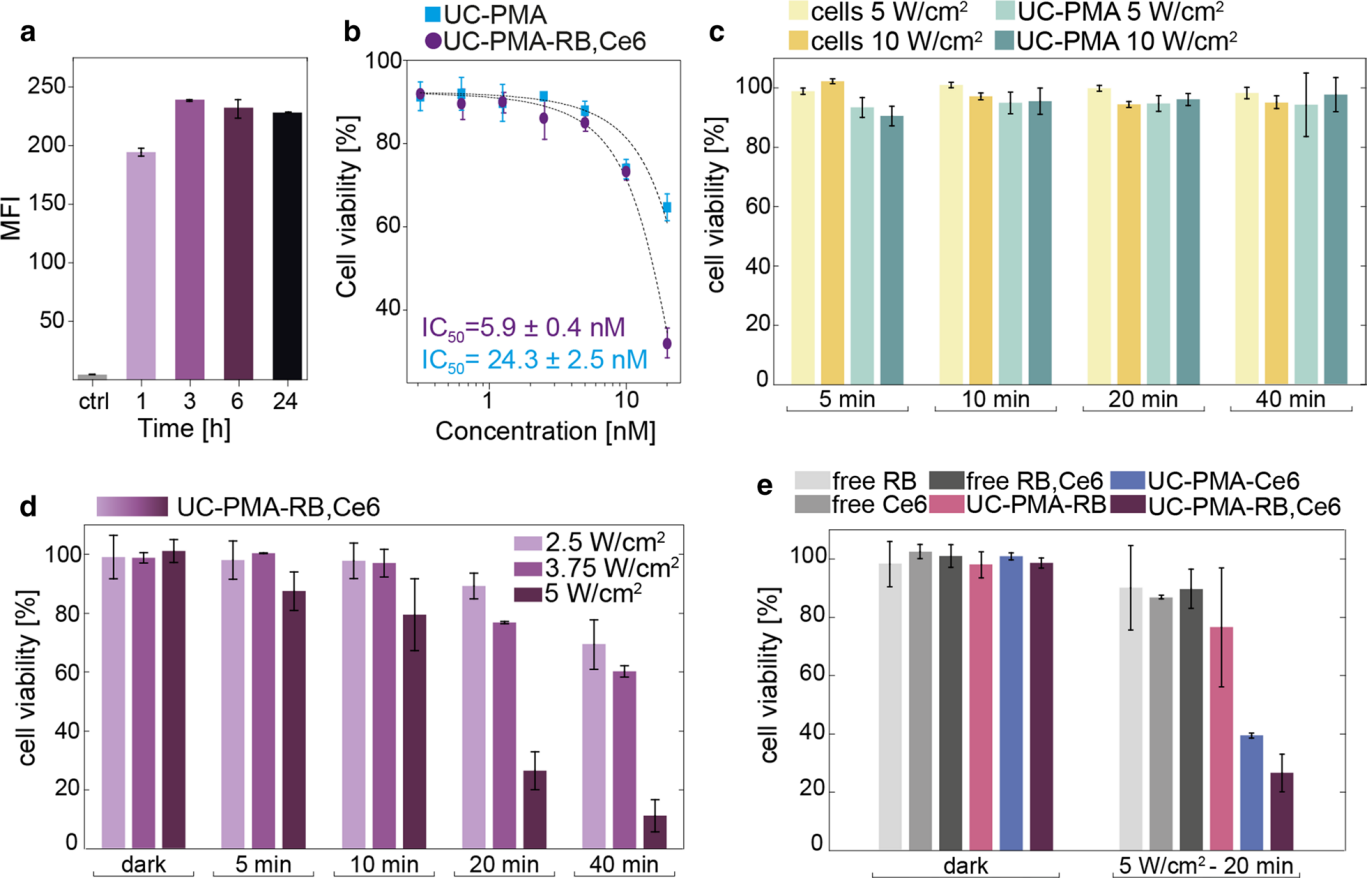
**a** Uptake of UC-PMA-RB,Ce6 (2 nM) by HeLa cells at different incubation times. **b** Viability studies of UC-PMA and UC-PMA-RB,Ce6 after 24 h of NPs incubation under dark conditions, and IC50 values. **c** Irradiation controls with untreated cells (only cells and laser) and cells loaded with UC-PMA (2.5 nM) after 3 h of incubation and different irradiation times using 5 or 10 W cm^−2^. **d** Impact on the cell viability of UC-PMA-PS (2.5 nM, 3 h of incubation) under different laser irradiances and different irradiation times. **e** Comparison in the viability impact of free PS and its combinations at a 250 nM, and the PS linked to the UCNPs incubated at 2.5 nM, under dark conditions, and after 20 min of irradiation with an 808 nm laser with a irradiance of 5 W cm^−2^

The in vitro behavior of the NPs was studied using different methodologies. First, the internalization rate of UC-PMA-RB,Ce6 NPs by HeLa cells was followed up from 1 to 24 h incubation (Fig. 3a). Interestingly, the internalization rate, as determined by the fluorescence of the RB attached to the NPs, remained constant since the first measured time point (3 h). In parallel, the internalization rate of UC-PMA NPs was also determined by ICP-MS after 3 h of incubation. The number of internalized NPs was ~ 13.4·10^3^ NP cell^−1^; this represents an internalization rate of ~ 0.05% of the total added NPs (see Additional file 1: Table S10). The order of magnitude of internalized cells is in good agreement with previous uptake experiments using PMA-coated NPs [45].

The viability under dark conditions in HeLa cells was studied after 24 h of exposure to the NPs (from 0.3 to 20 nM). Using a fitting function, the half maximal inhibitory concentration (IC50) value was determined, resulting in values in the range of previously reported IC50 for other PMA-coated NPs [41, 46]. As expected, the obtained IC50 value for the UC-PMA-RB,Ce6 NPs was lower than the value for the UC-PMA counterparts (Fig. 3b). These evidences support the fact that the UCNPs are not degraded in vitro, which would release toxic ions, and that the observed cytotoxicity is likely due to a combination of factors (e.g., size, organic shell, presence of dyes and their potential leaking, and number of particles per cell). Based on these results, the dose for cell-treatment with the UCNPs was fixed to 2.5 nM.

For all irradiation experiments a collimated laser beam with a controlled spot size was used, and therefore, each sample was homogeneously irradiated (Additional file 1: Figure S13). To establish the experimental conditions to perform the PDT therapy, irradiation experiments varying the irradiance (5 or 10 W cm^−2^) and the exposure time (from 5 to 40 min of irradiation) were performed with untreated HeLa cells and HeLa cells treated with UC-PMA NPs (2.5 nM) (Fig. 3c). In all the cases, the viability remained above 90%, showing that both the irradiances and exposure times with the 808 nm laser were harmless to cells, and that the UCNPs without PS do not impair cell viability, even under NIR irradiation.

Finally, PDT experiments were performed under strict dark conditions using UC-PMA-RB,Ce6 NPs at different laser irradiances (2.5, 3.75 and 5 W cm^−2^) and different irradiation times 5, 10, 20 and 40 min) using a constant NP concentration of 2.5 nM (Fig. 3d). Interestingly, the impact of the PDT in the cell viability was noticeable even with just 5 min of irradiation at the highest irradiance selected (5 W cm^−2^). Using that irradiance, viabilities below the 25% were reached following 20 min of irradiation. Viabilities close to the 50% were also obtained after 40 min of irradiation at 3.75 W cm^−2^; moreover, the lowest irradiance (2.5 W cm^−2^) had also an important effect in the viability with the longest irradiation time. It is important to notice that The American National Association of Standards in general recommends that a light fluency below 0.7 W cm^−2^ irradiated to the body can be considered as safe. Nevertheless, here there can be some controversy regarding which are the maximum accepted levels as occur, for example, for nanoparticle-based hyperthermia treatments, since there are not conclusive studies. For instance, recently Halas et al. reported a clinical pilot study regarding photothermal therapy for patients diagnosed with low- or intermediate-risk localized prostate cancer, in which they used 4.5–6.5 W laser sources coupled with optical fiber diffusers; such optical settings are in the same range than the ones here reported, and have obtained FDA approval for soft tissue ablation [47].

To confirm that the induced cell death was caused by an effective PDT, adequate controls were performed. These controls included the incubation of the cells with (i) the free PS, alone or combined, in a comparable dose to the one attached to 2.5 nM of UCNPs, i.e., 250 nM of PS(s), and (ii) the UCNPs with only one attached PS. The results summarized in Fig. 3e proved that none of the probes, including the UC-PMA-RB,Ce6 NPs, had any impact in the cellular viability if NIR light was not used. Also, after the PDT experiments, viabilities ~ 90% were found for all the cells treated only with the PS, alone or combined. Results proved that the most efficient PDT probe was, as expected, the UCNPs containing both PS (UC-PMA-RB,Ce6), followed by the UC-PMA-Ce6 (viability lower than 40%). Even though those experiments were performed in total dark conditions, during the course of this experiments, samples may suffer from some very short illumination coming from the cabin light, thus increasing the data variability showed in Fig. 3e.

**Fig. 4:**
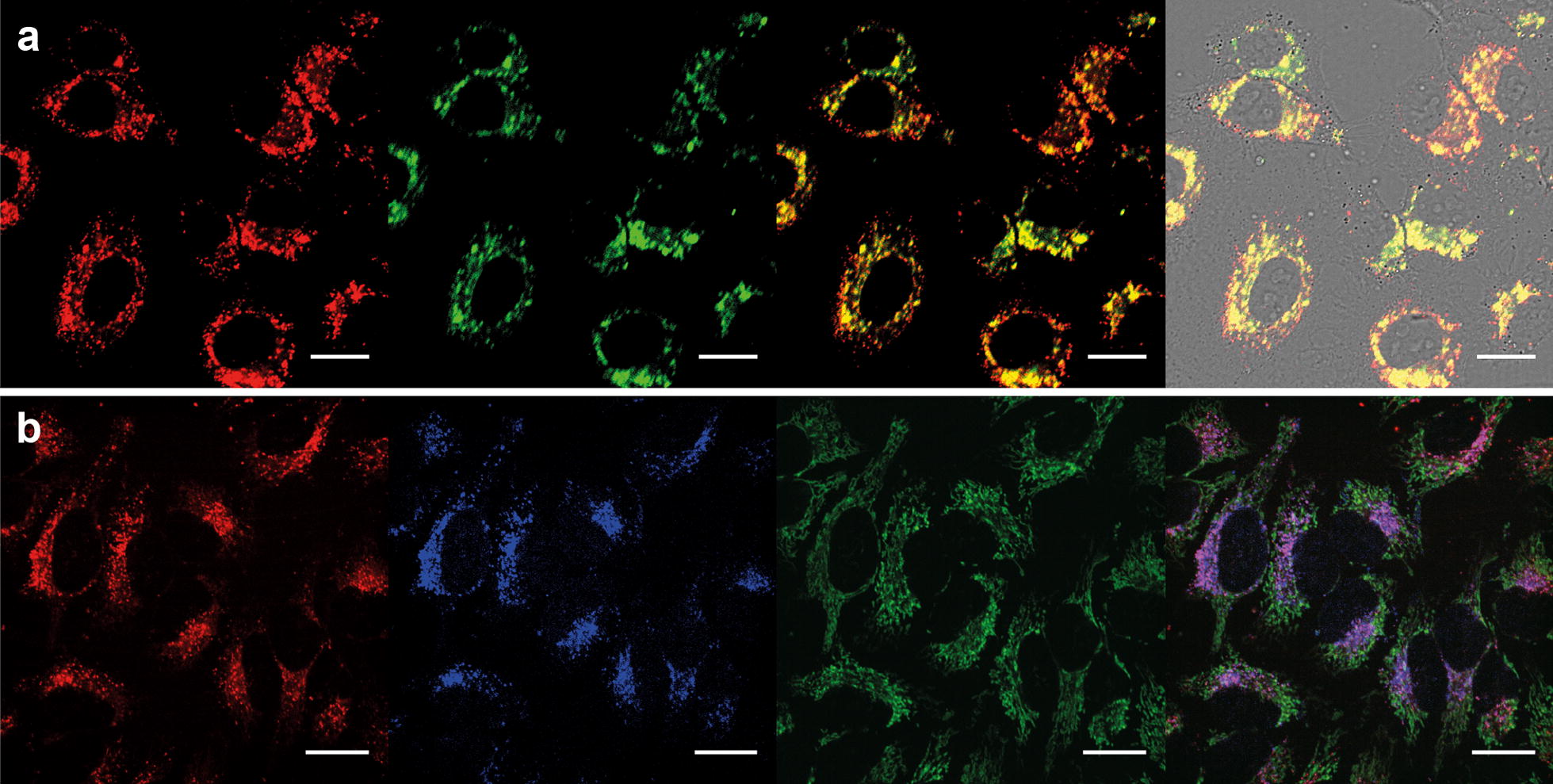
**a** Intracellular localization of UC-PMA-RB,Ce6 in HeLa cells after 3 h of incubation (2.5 nM of NPs). From left to right: Red (RB, Ex. 561, Em 620/60), Green (Ce6, Ex. 405, Em. 725/40), merged Red + Green (yellow color indicated colocalization of RB and Ce6), merged image of bright field (BF) + red + green. **b** Intracellular colocation of UC-PMA-RB and lysosomes and mitochondria in HeLa cells after 3 h of incubation. From left to right: Red (RB, Ex. 561, Em 620/60), Blue (Lysotracker Blue, Ex. 405, Em. 450/50), Green (Mitotracker Green, Ex. 488, Em. 525/50), merged image of blue + red + green. Purple indicates lysosomes and UCNPs colocalization. Scale bars represent 20 µm

After proving the efficiency of the developed UCNPs as 808 nm-PDT agents, next step was evaluating their intracellular location and study the mechanism by which the PDT occurs. Therefore, the internalization location of UC-PMA NPs was studied. Confocal microscopy images of HeLa cells incubated for 3 h with UC-PMA-RB,Ce6 NPs (2.5 nM) showed that both PSs were colocalized intracellularly (100× and 60× magnification images are shown in Fig. 4a and Additional file 1: Figure S14, respectively). As expected, the NPs were distributed in the perinuclear region indicating that the NPs are likely to be storaged in lysosomal compartments (see z-scan slices in Additional file 1: Figure S15). This intracellular location has been reported previously for similar systems involving PMA-coated NPs [48]. Colocalization experiments were performed to confirm this evidence. To do so, lysosomes and mitochondria of HeLa cells treated with UC-PMA-RB NPs were stained with Lysotracker and Mitotracker dyes, respectively. As expected, the intracellular location of the UCNPs was colocalized with the lysosome staining but not with the mitochondria one (Fig. 4b and Additional file 1: Figure S16).

**Fig. 5:**
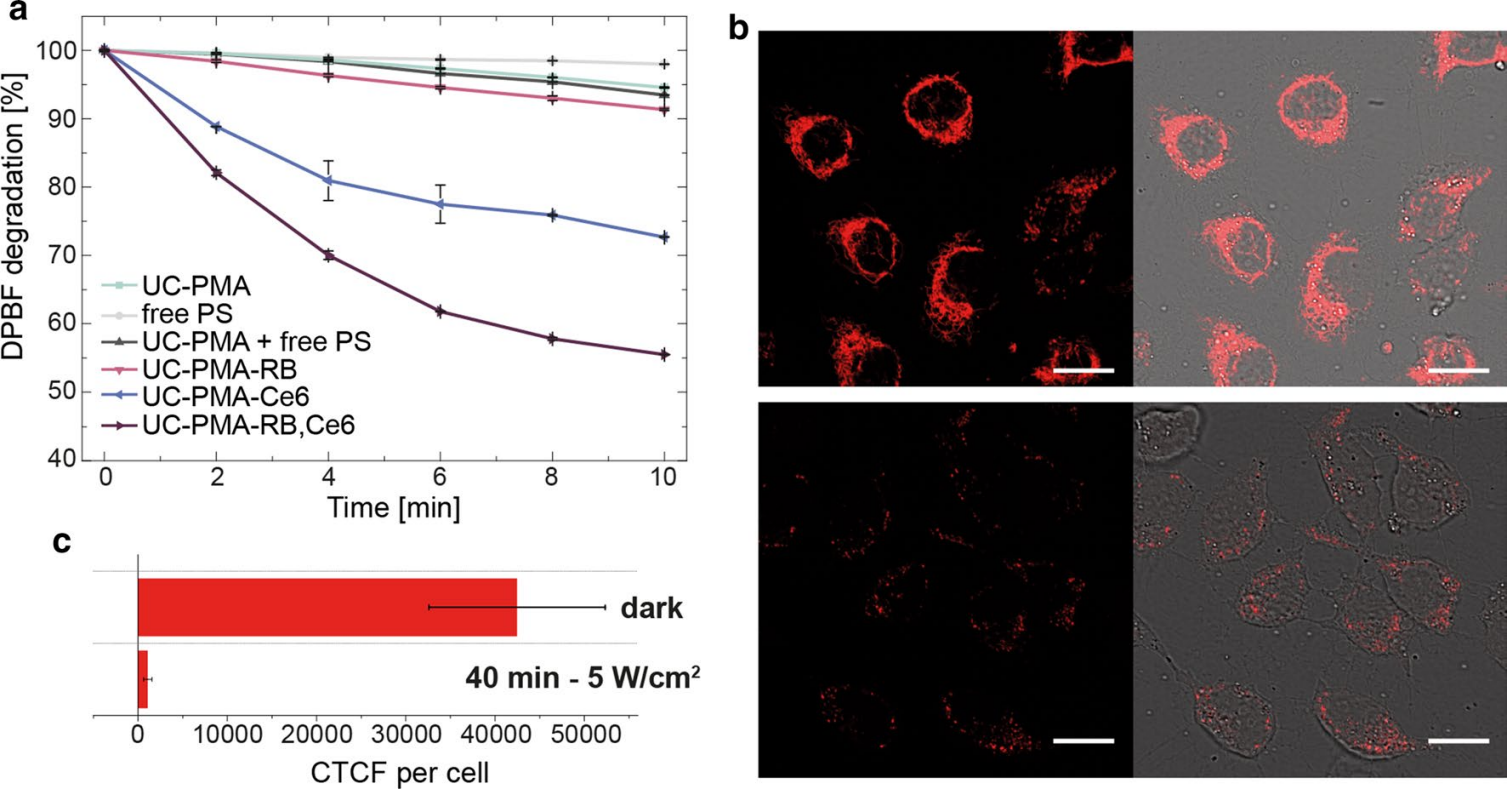
**a** Degradation rate of DPBF at 407 nm upon 5 W cm^−2^ irradiation with an 808 nm laser at 2.5 nM concentration of UCNPs or 250 nM of free PS. **b** Cells treated with UC-PMA-Rb,Ce6; 2.5 nM for 3 h under dark conditions after TMRE exposure (upper raw), and after 40 min of 5 W cm^−2^ irradiation (lower raw). **c** Corrected-total cell fluorescence (CTCF) of cells incubated for 3 h with 2.5 nM UC-PMA-RB,Ce6 and TMRE with and without PDT

To conclude, the production of ROS was studied both in test tube and in vitro. First, the degradation rate of 1,3-diphenylisobenzofuran (DPBF) was followed over time (2 min) under a constant irradiation of 5 W cm^−2^ with the 808 nm laser irradiation setup (Fig. 5 and Additional file 1: Figure S17 for raw data). The degradation of this probe is due to the ROS production in solution, which leads to a loss of absorbance at 407 nm. Free PSs, and all types of UCNPs were tested under the same conditions. Results are summarized in Fig. 5a, which clearly shows a higher degradation rate of DPBF in the presence of UC-PMA-RB,Ce6 NPs. This trend was followed by the UCNPs modified with Ce6. Lower degradation rates were observed for UC-PMA-RB NPs, and UC-PMA NPs mixed with free PS or alone, respectively. The irradiation of the PS alone does not induce any significant DPBF degradation. The trend observed in the test tube experiment correlates perfectly with the observed trend in the induced cell toxicity assay, which supports that the cell death is induced by ROS production.

Finally, the intracellular ROS production was determined using a mitochondrial membrane potential assay based on the use of a tetramethylrhodamine ethyl ester (TMRE). This probe can label active mitochondria but cannot label depolarized or inactive mitochondria. Therefore, if upon PDT mitochondria are impaired, the labelling with TMRE will be reduced. Figure 5b, c shows the differences in TMRE labeling of mitochondria in cells incubated with UC-PMA-RB,Ce6 NPs before and after NIR exposure (for additional images see Additional file 1: Figure S18). A clear decrease of fluorescence is observed (Fig. 5b) after the exposure to the 808 nm light indicating that the mitochondria potential is affected by the ROS produced by the UC-PMA-RB,Ce6 NPs. This decrease in fluorescence was quantified using a fluorescence readout, that is, the corrected total cell fluorescence (CTCF) per cell, before and after irradiation; the differences are clear, dropping from a CTCF per cell ~ 40,000 before irradiation to a CTCF close to 1000 after irradiation (Fig. 5c).

## Conclusions

In this work, we have prepared a modification of the methanol-assisted high temperature UCNP synthetic process and subsequent layer-by-layer functionalization methodology to produce highly monodisperse 808-active UCNPs. These particles have been water transferred using a DBCO-modified amphiphilic polymer that allows the efficient linkage of two different PSs by click chemistry. The PS selection was done in order to maximize the absorbance of the emitted light from the UCNPs. The UC-PMA-PS(s) nanoplatforms have been tested as PDT agents in vitro showing a good performance, and a great colloidal stability even in complex biological media. The ability of these UCNPs to produce ROS in test tube and in vitro has been demonstrated. Once intracellularly stored in lysosomal compartments, the produced ROS are able to act in the mitochondria.

## Materials and methods

### General considerations

All the reagents including 1-octadecene (ODE, 90%), oleic acid (OA, 90%), ethanol (EtOH) and methanol (MeOH), dimethylformamide (DMF), chloroform, milli-Q water, tetrahydrofuran (THF), yttrium(III) chloride anhydrous (YCl_3_, 99.9%), ytterbium(III) chloride anhydrous (YbCl_3_, 99.9%), erbium(III) chloride anhydrous (ErCl_3_, 99.9%), ammonium fluoride (NH_4_F, 96%), Sodium hydroxide (NaOH, 96%), Sodium trifluoroacetate (Na-TFA, 98%), neodymium(III) chloride (NdCl_3_ 99.9%), 1-ethyl-3-(3-dimethylaminopropyl)carbodiimide (EDC), N-hydroxysuccinimide (s-NHS), Rose Bengal (RB), 6-bromohexanoic acid (Br-HA), poly(isobutylene-alt-maleic anhydride), dodecylamine (DDA), dibenzocyclooctine-amine (DBCO-NH_2_), 1,3-Diphenylisobenzofuran (DPBF, 97%) were purchased from Sigma-Aldrich. Chlorin e6 (Ce6) was purchased from Santa Cruz Biotechnology. 3-azido-propylamine (NH_2_-N_3_) was purchased from Fluorochem Ltd.

### Synthesis of 980 nm activable cores

The synthesis of highly monodisperse β-NaYF_4_:Yb_18%_Er_2%_ was carried out by modification of previously reported methods [49, 50]. Typically, 2 mmol of precursors at the desired proportions (80% of YCl_3_ or 485.34 mg; 18% of YbCl_3_ or 139.50 mg and 2% ErCl_3_ or 15.27 mg) were mixed into a 250 mL three-neck round bottom flask with 30 mL of ODE and 12 mL of OA. The solution was heated slowly (3 °C min^−1^) to 150 °C under vacuum to eliminate oxygen and water traces. After 1 h, the solution was cooled down and subsequently, 296.5 mg of NH_4_F and 200 mg of NaOH dissolved in 10 mL of methanol were added to the flask and kept under stirring during 30 min at 50 °C. A translucent yellow-white solution was formed. Then, methanol was evaporated under vacuum and by increasing the temperature to 80 °C. After 20 min, the solution became transparent. Finally, the reaction was heated at 7 °C min^−1^ up to 300 °C under N_2_ atmosphere for 1 h. The cores were precipitated with methanol (20 mL) and isolated by centrifugation (2000 RCF, 10 min). The purified cores were dispersed in 30 mL of chloroform, ca. 500–600 mg per synthesis were obtained. The cores were characterized by electron microscopy techniques and ICP-MS. The final concentration of the solution was determined by combining the results from TEM and ICP-MS.

### Preparation of shell precursors

The solutions needed for shell growing onto the previously synthesized cores were prepared before starting with the final reaction [10, 33]. Na-TFA-OA was prepared by mixing 10 mL of OA and 4 mmol of Na-TFA under vacuum at room temperature to obtain a final solution of 0.4 M. Ln-OA solutions were prepared with the lanthanide elements in the desired proportions. Two different precursors were prepared (Y_90%_,Yb_10%_-OA and Nd_90%_,Yb_10%_-OA) by mixing 10 mL of OA, 15 mL of ODE and 2.5 mmol of precursors mixture and heating the solution to 150 °C under vacuum. In this way, for Y,Yb-OA, we added 2.25 mmol (682.56 mg) of YCl_3_ and 0.25 mmol (96.87 mg) of YbCl_3_ and for Nd,Yb-OA we mixed 2.25 mmol (563.85 mg) of NdCl_3_ and 0.25 mmol (96.87 mg) of YbCl_3_.

### Synthesis of 808 nm switchable core @shell_1_ @shell_2_ @shell_3_ upconverting nanoparticles (UCNPs)

The selected structure was NaYF_4_:Yb_18%_Er_2%_ @NaYF_4_:Yb_10%_ @NaNdF_4_:Yb_10%_ @NaYF_4_:Yb_10%_. 2 mL of a 600 nM solution of the previously purified cores in chloroform (1.2 nmol of cores) were mixed with 4 mL of OA and 6 mL of ODE into a 100 mL three-neck round bottom flask. Solution was heated to 50 °C and kept under vacuum to evaporate the chloroform. After this, reaction was heated quickly (10 °C min^−1^) to 300 °C and kept under N_2_ flux. Once the temperature was stabilized, precursor solutions were added to the reaction flask. The reaction time after each injection was 15 min. Finally, reaction was cooled down to room temperature and NPs were collected by centrifugation (2000 RCF, 5 min) after adding 10 mL of ethanol. The supernatant was discarded and a solution at 600 nM NPs was obtained by adding 2 mL of chloroform to the pellet. This process was repeated to grow the desired shell number following the steps.

### Carboxylate Rose Bengal (RB-COOH)

The modification of the commercial Rose Bengal was done following a previous protocol [15]. RB and Br-HA were mixed in a 1:1 molar ratio. For that, two solutions in DMF were prepared, one containing 101.8 mg of RB dissolved in 2 mL of DMF and a second one containing 19.5 mg of Br-HA dissolved in 2 mL of DMF at 70 °C until both solutions are completely dissolved. Then, the RB solution was added onto the Br-HA solution and stirred at 70 °C for 24 h. Final product was dried and stored under dark conditions.

### N_3_-modification of photosensitizers

The two carboxylated-photosensitizers (PS) (RB-COOH and commercial Chlorin e6 (Ce6)) were activated with carbodiimide. In general, 400 µL of RB-COOH or Ce6 (1.8 mM in MeOH) were added to 2 mL of DMSO. 800 µL of EDC (3 mg/mL in DMSO) and 400 µL of sulfo-NHS (5 mg/mL in DMSO) freshly prepared were added to activate the carboxylic groups of the PSs. After 30 min of stirring at room temperature and dark conditions, 144 µL of the ligand NH_2_-N_3_ was added (10.2 mM in DMSO). The reaction was left overnight, and final solutions were kept at 4 °C and under dark conditions for further use, considering the final concentration of both PS-N_3_ as 192 µM in DMSO. The PS’s concentration was determined using the corresponding calibration curves (Additional file 1: Figure S9).

### DBCO-modified polymer preparation

The amphiphilic polymer dodecyl grafted-poly(isobutylene-alt-maleic-anhydride) (PMA) was synthesized as described previously [51] with some modifications. Briefly, 1.85 mmol of poly-isobutylene-alt-maleic-anhydride (285 mg, M_w_ ≃ 6000 g mol^−1^), 1.39 mmol of DDA (265.8 mg, 75% of the monomers) and 0.04 mmol of DBCO-NH_2_ (2% of the monomers) (10.2 mg) were mixed together in 50 mL of THF. The solution was kept under magnetic stirring and reflux overnight. THF was evaporated and the dried polymer was dispersed with 3.7 mL of chloroform to obtain a final monomer concentration of 0.5 M. An equivalent protocol without DBCO was followed to produce plain PMA, referred as PMA (bare).

### PMA coating

The procedure was performed by mixing 100 µL of UCNPs in chloroform at ~ 300 nM with 150 µL of PMA(bare) or PMA (with DBCO) 0.25 M and 1 mL of chloroform. After this, the solvent was evaporated under vacuum in a round bottom flask with a rotavapor system. Then, 2 mL of sodium borate buffer (SBB, 0.1 M, pH 12) were added. Samples were sonicated until a transparent and colorless solution was obtained. NPs were centrifugated at 30,000 RCF during 30 min at 10 °C. Final solutions of UCNP@PMA were kept in milli-Q water and characterized.

### PS-N_3_ and UC-PMA NPs conjugation by click chemistry

The modification with one or two PS of UC-PMA NPs was performed using a copper-free click chemistry reaction. Typically, 500 µL of UC-PMA at 60 nM (particles concentration) in water were mixed with RB-N_3_ and Ce6-N_3_ vigorously for 30 min. The ratio of dye per UCNP was fixed to 750 (for each PS). Three different PDT (PDT) platforms were prepared, (i) RB only (NP:RB 1:750), (ii) Ce6 only (NP:Ce6 1:750) and (iii) with RB and Ce6 (NP:RB 1:750 and NP:Ce6 1:750). When reaction was finished, samples were dialyzed overnight throw a membrane of MWCO 14,000 kDa to remove all non-covalently bond molecules. Finally, samples were precipitated (7000 RCF, 10 min, 10 °C) and dispersed in water to yield solutions of 300 nM. The PS number and concentration were determined using independent fluorescence calibration curves (Additional file 1: Figure S9).

### Stability studies in biological media

The stability of the polymer-coated nanoparticles with and without PS in different media overtime was studied. Samples were incubated in water, artificial lysosomal fluid (ALF), ALF supplemented with 10% of FBS and complete cell culture medium (Dulbecco’s Modified Eagle Medium, DMEM, supplemented with 10% of FBS) under stirring up to a week.

#### DPBF degradation

In order to perform the experiment, 1 mL of a freshly prepared solution of the sample of interest was mixed with 10 µL of a freshly prepared DPBF solution (10 mM ethanol). Samples were maintained under dark conditions, in 1 cm path length Hellma quartz cells inside an UV–visible spectrophotometer (Biochrom Libra S60-UV) equipped with an 808 nm laser. Samples were irradiated at periods of 2 min at 5 W cm^−2^ from the top and the absorption value at 407 nm was recorded. UC-PMA-RB, UC-PMA-Ce6, UC-PMA-RB,Ce6 at a concentration of 2.5 nM in water. As controls, UC-PMA (2.5 nM), free dyes RB and Ce6 (250 nM) and 2.5 nM of UC-PMA mixed with free dyes RB-N_3_ and Ce6-N_3_ at 250 nM.

### Cell culture

HeLa (human cervical cancer cell line) were cultured in Dulbecco’s Modified Eagle Medium with phenol red, 4.5 g L^−1^d-glucose, l-glutamine and pyruvate (DMEM, 1X, Gibco) supplemented with 10% FBS (Gibco) and 1% Penicillin/Streptomycin (P/S, Corning, 100X). Cells were maintained under humid conditions at 37 °C and 5% of CO_2_. Cells were passaged after cleaning Dulbecco’s Phosphate Buffered Saline (DPBS, 1X, Gibco) with 0.25% Trypsin–EDTA (1X, Gibco) when the culture reached confluency.

### Laser irradiation experiments

Cells were seeded in multi-well plates and an 808 nm laser (Lasing, #FC-W-808A) implemented to a zoom fiber collimator (Thorlabs, #ZC618SMA) was used to perform all irradiations. To obtain the spot size an infrared viewing card (Thorlabs, #VRC4) was used to see the spot and ImageJ to measure it. As the beam is collimated (see fabricant details for laser collimation conditions), a homogeneous spot is produced and thus, the irradiance can be easy calculated just dividing the power by the spot surface (in cm^2^). Different time and irradiance conditions (as well as spot sizes) were performed as it is indicated in each experiment procedures.

### Nanoparticles internalization studies

#### ICP-MS

Typically, 250,000 cell/well were seeded in 2 mL of media in 6-well plates. After 24 h, media was removed and 2 mL of 5 nM of UC-PMA dispersed in media, were added. After 3 h of cell exposure to the UCNPs, media was removed, and cells were washed with PBS (× 3). 200 µL of trypsin were added and cells were collected with 1 mL of DMEM and counted after that. After cell centrifugation (5000 RPM, 6 min), they were digested overnight in aqua regia (200 µL). Then, they were diluted with 4.8 mL of HCl 2% for ICP-MS analysis. The ICP-MS analysis was performed in an Agilent 7700x inductively coupled plasma mass spectrometer.

#### Cytometry studies

15,000 cell/well were seeded in 48 well plates in 200 µL of complete cell media. 24 h later, 2 nM of previously diluted UC-PMA-RB,Ce6 in media were added to the cells and kept 1, 3, 6, 12 and 24 h under incubation. After three washes with PBS, cells were treated with 50 µL of trypsin and diluted with 150 µL of PBS (supplemented with 10% FBS) and analyzed by cytometry in a Guava^®^ easyCyte BG HT flow cytometer (Millipore^®^). Cell fluorescence was collected in the Yel-G channel (Ex. 532 nm, Em. 583/26 nm) counting always at least 5000 events. Controls were also performed with cells without UCNP treatment.

#### Confocal imaging of living cells

Typically, 20,000 HeLa cells in 200 µL were seeded on µ-Slide 8 well-ibiTreat chambers (1 cm^2^*per* well, Ibidi, Germany) at least 12 h before the particle exposure. UCNPs were diluted to a concentration of 2.5 nM in cell media. After 3 h, cells were cleaned with PBS (3×) in order to remove non-associated particles. 200 µL of supplemented DMEM without phenol red were added to the cells before imaging on an Andor Dragonfly spinning disk confocal system mounted on a Nikon TiE microscope equipped with a Zyla 4.2 PLUS camera (Andor, Oxford Instruments) and an OKO-lab incubator to keep cells at 37 °C during experiment time. RB channel was obtained with an excitation of a 561 nm laser and a 620/60 nm filter. Ce6 emission was collected exciting with a 405 nm laser and using a 725/40 nm filter. All the images were processed with the free software ImageJ. For colocalization experiments lysosomes were labeled with lysotracker blue (Ex. 405, Em. 450/50, Thermofisher) and Mitotracker Green FM (Ex. 488, Em. 525/50, Thermofisher) following the manufacturer instructions.

### Cell viability

Resazurin assay was performed on HeLa cells seeded in 96-well plates (NEST Scientific). In general, 7500 cells per well in 100 µL of cell medium 24 h before the UCNPs exposition. Then media was removed and 100 μL of cell medium with the desired concentration of UCNPs were added and incubated at 37 °C and 5% CO_2_. After the desired time, each well was washed with PBS (3×) and 100 µL of a freshly prepared 10% resazurin solution in cell media (resazurin sodium salt in water 0.2 mg mL^−1^ filtered; Resazurin Sodium Salt, Sigma Aldrich) were added per well. After 3 h, fluorescence intensity was measured with a plate reader (Infinite^®^ 200 PRO, Tecan, Switzerland) under 560/20 nm excitation and 610/20 nm emission filter. The value for control cells (${\text{I}}_{\text{C}} = {\text{I}}_{ + } - {\text{I}}_{ - }$), untreated cells, is an average of at least nine independent well values. Sample values (${\text{I}}_{\text{S}}$) are a mean of three independent well values. Cell viability values are calculated as follow:

$${\text{cell viability }}\left[ \% \right] { = }\frac{{{\text{I}}_{\text{sample}} - {\text{I}}_{ - } }}{{{\text{I}}_{\text{C}} }} \cdot 1 0 0$$

### Photodynamic therapy

7500 cell/well in 100 µL were seeded in 96 well plates. After 24 h, cells were exposed to a fresh solution of 2.5 nM of UCNPs in complete cell media. After 3 h, media was removed, and cells were washed with PBS three times. Then, 100 µL of clean media supplemented with HEPES and without phenol red were added. Laser irradiations were performed at different times and powers with a spot size of 0.33 cm^2^. Cells were kept in dark conditions, humidity and 37 °C during the irradiation time using an incubator. Then, cells were incubated overnight. Afterwards, cell viability was read using the resazurin viability test. The four platforms were tested at a concentration of 2.5 nM: UC-PMA (as control), UC-PMA-RB, UC-PMA-Ce6 and UC-PMA-RB,Ce6.

### ROS production measurements

#### Confocal microscopy TMRE studies

Confocal imaging experiments were done comparing cells without laser treatment and after 40 min of exposure to 5 W cm^−2^ using a protocol similar to the one performed for confocal internalization studies. After the incubation with the UCNPs, cleaning and subsequent irradiation, cells were treated with a 100 nM TMRE solution for 30 min. Cells were maintained under dark, temperature and humidity conditions in all the steps of the experiment, even during acquisition.

## Supplementary information

**Additional file 1.** General considerations: Synthesis of 808 nm-activable core-multishell upconversion nanoparticles; Characterization of 808 nm-activable upconversion core-multishell UCNPs; Synthesis and characterization of water dispersable UCNPs after click chemistry onto the surface; Photodynamic therapy activation by 808 nm irradiation and ROS production.
