# Supplementary material for: 808 nm-activable core@multishell upconverting nanoparticles with enhanced stability for efficient photodynamic therapy
Source: J Nanobiotechnology. 2020 Jun 5;18:85. doi: 10.1186/s12951-020-00640-3 (PMC7275415; doi:10.1186/s12951-020-00640-3)
Supplement: Supplementary file 1 — Additional file 1. General considerations: Synthesis of 808 nm-activable core-multishell upconversion nanoparticles; Characterization of 808 nm-activable upconversion core-multishell UCNPs; Synthesis and characterization of water dispersable UCNPs after click chemistry onto the surface; Photodynamic therapy activation by 808 nm irradiation and ROS production. [file 12951_2020_640_MOESM1_ESM.pdf]

808 nm-activable core@multishell upconverting nanoparticles with enhanced stability for efficient photodynamic therapy

Raquel Martínez[1,2], Ester Polo[1,2], Silvia Barbosa[2,3], Pablo Taboada[2,3], Pablo del Pino[1,2]* and Beatriz Pelaz[1,4]*

[1] Centro Singular de Investigación en Química Biolóxica e Materiais Moleculares (CiQUS), Universidade de Santiago de Compostela, Santiago deCompostela 15782, Spain

[2] Grupo de Física de Coloides y Polímeros, Departamento de Física de Partículas, Universidade de Santiago de Compostela, Santiago de Compostela 15782, Spain.

[3] Instituto de Investigaciones Sanitarias, Universidade de Santiago de Compostela, Santiago de Compostela 15782, Spain.

[4] Grupo de Física de Coloides y Polímeros, Departamento de Inorgánica, Universidade de Santiago de Compostela, Santiago de Compostela 15782, Spain.

* correspondence: pablo.delpino@usc.es, beatriz.pelaz@usc.es

Additional file 1

Table of Contents

I. General considerations. **2**

II. Synthesis of 808 nm-activable core-multishell upconversion nanoparticles **2**

III. Characterization of 808 nm-activable upconversion core-multishell UCNPs **3**

IV. Synthesis and characterization of water dispersable UCNPs and surface modification **8**

a) Polymer synthesis and previous modification of photosensitizers 8

b) PMA functionalization of oleic acid caped UCNPs 9

c) Copper free click chemistry onto the UC-PMA 11

d) Characterization of UCNP nanoplatforms 12

V. Photodynamic therapy activation by 808 nm irradiation and ROS production **15**

I. General considerations.

All the reagents including 1-octadecene (ODE, 90%), oleic acid (OA, 90%), chloroform, ethanol (EtOH) and methanol (MeOH), dimethylformamide (DMF), milli-Q water, tetrahydrofuran (THF), yttrium(III) chloride anhydrous (YCl_3, 99.9%), ytterbium(III) chloride anhydrous ($YbCl_3$, 99.9%), erbium(III) chloride anhydrous ($ErCl_3$, 99.9%), ammonium fluoride (NH_4F, 96%), sodium hydroxide (NaOH, 96%), sodium trifluoroacetate (Na-TFA, 98%), neodymium(III) chloride ($NdCl_3$ 99.9%), 1-ethyl-3-(3-dimethylaminopropyl)carbodiimide (EDC), n-hydroxysuccinimide (s-NHS), Rose Bengal (RB), 6-bromohexanoic acid (Br-HA), poly(isobutylene-alt-maleic anhydride) (PMA), dodecylamine (DDA), dibenzocyclooctine-amine1 (DBCO-NH_2), 1,3-Diphenylisobenzofuran (DPBF, 97%) were purchased from Sigma-Aldrich. Chlorin e6 (Ce6) was purchased from Santa Cruz Biotechnology. 3-azido-propylamine (NH_2-N_3) was purchased from Fluorochem Ltd.

II. Synthesis of 808 nm-activable core-multishell upconversion nanoparticles

Synthesis of 808 nm-active core@shell$_1$@shell$_2$@shell$_3$ upconverting nanoparticles (UCNPs). The injection sequence and volumes used to grow the shells are summarized in Table S1. Figure S1 shows the experimental setup.

**Shell$_1$**	$NaYF_4$:$Yb_{10\%}$	1 mL of Y,Yb-OA → 0.5 mL of Na-TFA-OA →1 mL of Y,Yb-OA → 0.5 mL of Na-TFA-OA
**Shell$_2$**	$NaNdF_4$:$Yb_{10\%}$	1 mL of Nd,Yb-OA→ 0.5 mL of Na-TFA-OA→1 mL of Nd,Yb-OA → 0.5 mL of Na-TFA-OA
**Shell$_3$**	$NaYF_4$:$Yb_{10\%}$	1 mL of Y,Yb-OA → 0.5 mL of Na-TFA-OA →1 mL of Y,Yb-OA → 0.5 mL of Na-TFA-OA

Table S1. Sequence of reactants injections.

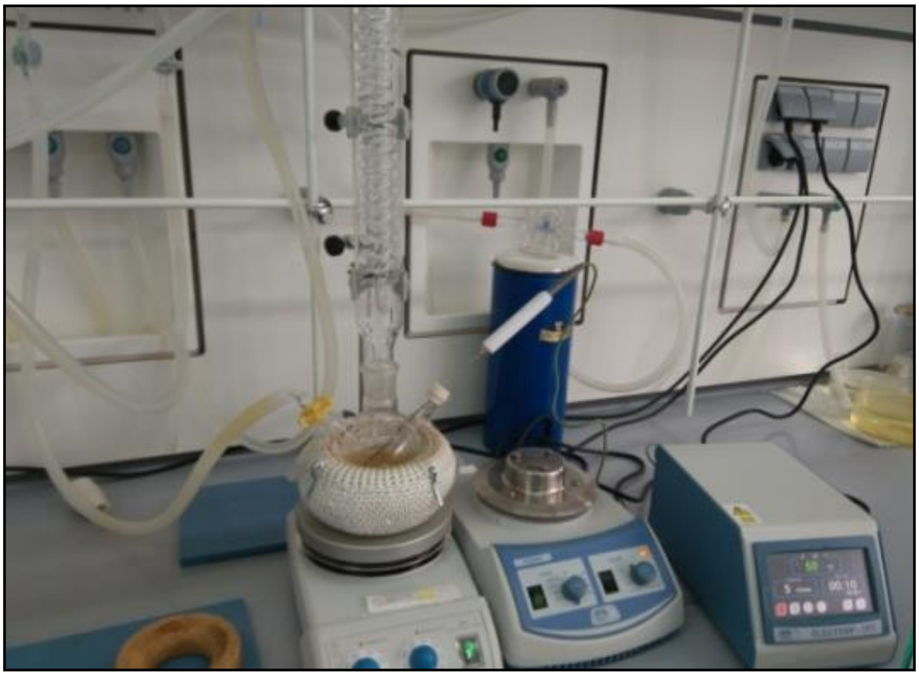

Figure S1. Experimental setup for UCNP´s synthesis

III. Characterization of 808 nm-activable upconversion core-multishell UCNPs

Scanning and transmission electron microscopy (SEM and TEM). SEM images were acquired with a FESEM Zeiss Ultra Plus operated at 3 kV or 20 kV. TEM micrographs were obtained using a JEOL JEM-2010 electron microscope operated at an accelerating voltage of 120 kV. For SEM images, 5 µL of diluted samples were deposited onto a silicon wafer before air-drying. In order to prepare TEM samples, 3 µL of a diluted sample was deposited on a carbon film supported on a 400-mesh copper grid (Electric Microscopy Sciences, CF400-CU) and was air-dried. Results confirmed that core and core-shell structures were perfectly monodispersed and all shells growth uniformly around the cores, keeping NPs morphology almost intact and producing a single population.

SEM and TEM micrographs from core and core@shell$_1$@shell$_2$@shell$_3$ structures are shown in Figures S2-S3. Using the free ImageJ software, the diameter (*d*) of at least 300 particles was measured and histograms were plotted.

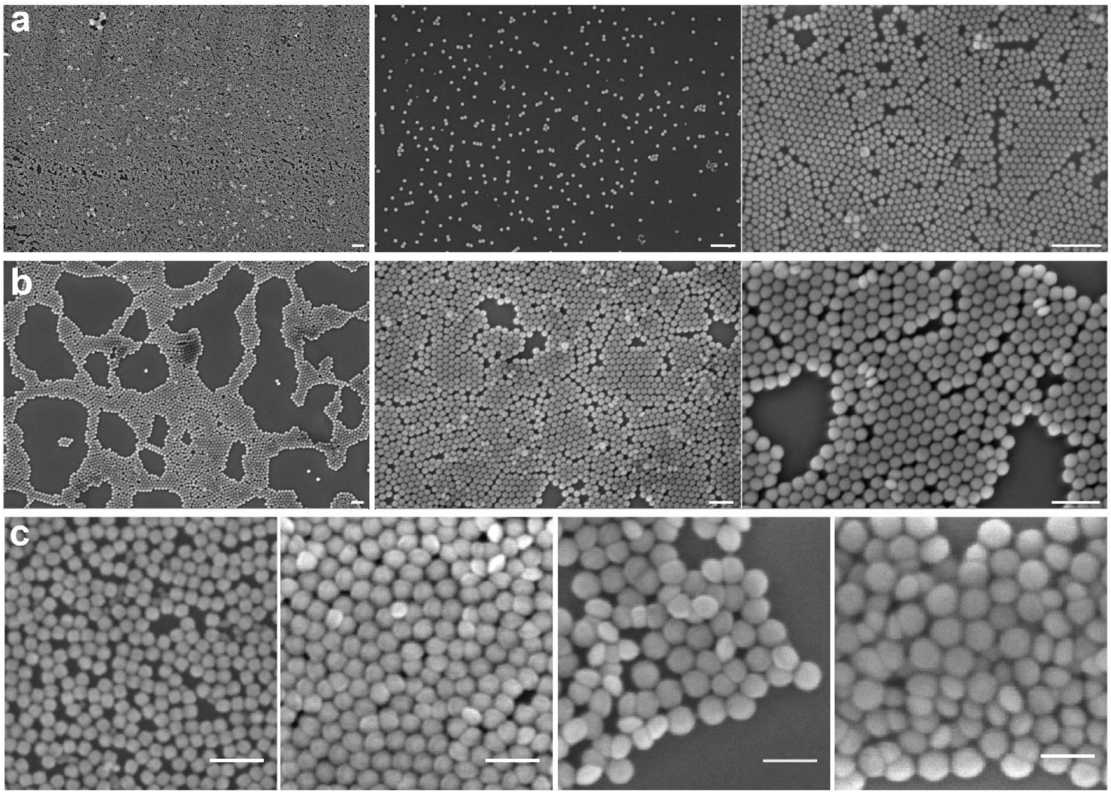

Figure S2. a,b) SEM micrographs at different magnifications (50 kX, 100 kX and 200 kX) for cores (a) and core@multishell (b) structures. Scale bars represent 200 nm. c) SEM micrographs from the different growing steps. From left to right, core, core@shell$_1$, core@shell$_1$@shell$_2$ and core@shell$_1$@shell$_2$@shell$_3$. Scale bars represent 100 nm.

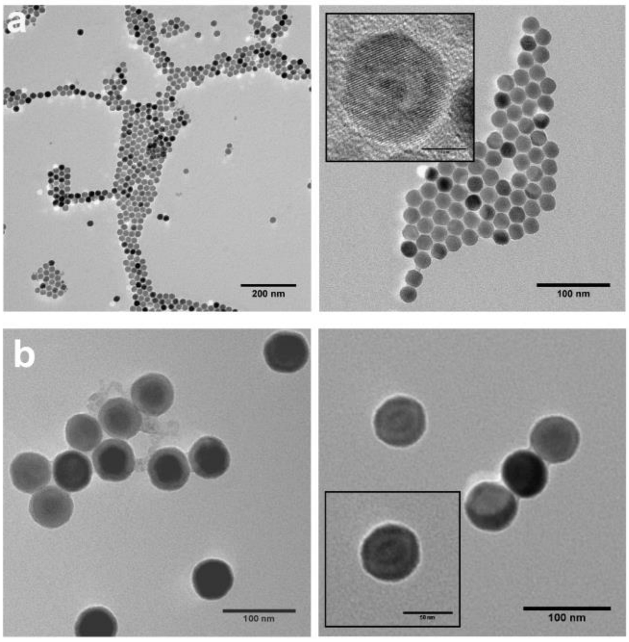

Figure S3. TEM micrographs at different magnifications (50 kX, 100 kX and 200 kX) for a) 980 nm-UC cores and b) 808 nm-UC core@$shell_1$@$shell_2$@$shell_3$ structure.

<u>*Powder X-ray diffraction.*</u> Powder X-ray analysis were carried out in an X-ray diffractometer Philips to study the crystalline powder of samples; the samples were examined in the range of 2θ between 2° and 75°, with a passage of 0.02° and a time by step of 2s. The UCNPs cores showed a good correlation (red peaks) with the standard pattern for $NaFY_4$, (ICDD PDF #16-0334) which confirm the cores hexagonal crystalline structure. In Table S2 there is a list of the identified peaks in the cores sample.

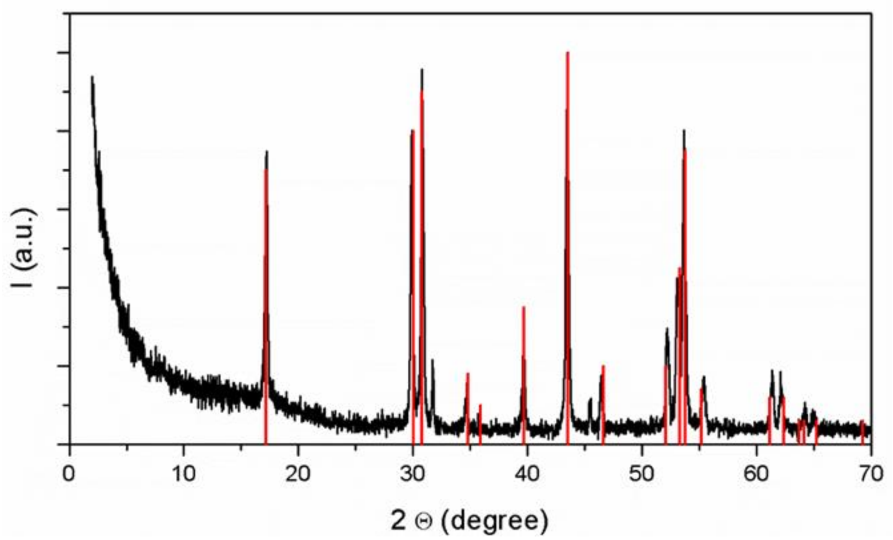

Figure S4. X-ray spectra from core UCNPs showing its hexagonal structure and the overlapping with the $NaYF_4$ standard pattern structure.

Pos. [°2Θ.]	d-spacing [Å]	Rel. Int. [%]	Height [cps]	Struct. FWHM [°2Θ]
17.183(3)	5.15646	66.57	84.99	0.159763
29.876(2)	2.98824	81.86	104.52	0.139381
30.796(2)	2.90106	91.94	117.39	0.152761
31.707(5)	2.81981	17.37	22.18	0.077344
34.67(1)	2.58553	11.95	15.26	0.126266
39.608(6)	2.27360	25.17	32.13	0.156949
43.436(2)	2.08166	100.00	127.68	0.163850
45.421(9)	1.99522	11.65	14.88	0.075171
46.394(7)	1.95562	15.97	20.39	0.123563
52.167(5)	1.75195	30.77	39.29	0.152546
53.062(5)	1.72448	41.66	53.19	0.173181
53.625(4)	1.70770	87.82	112.13	0.151593

Table S2. List of major peaks identified in the cores.

Inductively coupled plasma mass spectrometry (ICP-MS). ICP-MS analysis was performed in order to determine the final concentration and composition of the NPs after and during the synthesis and the dopants percentage in each of the core or shells.

Three different and independent core synthesis were analyzed via ICP-MS. Samples obtained during the shell growing steps from the same synthetic process were analyzed in order to determine the individual shell composition.

20 µL of all the samples were digested overnight with 480 µL of aqua regia. Samples were diluted with 4.5 mL of HCl 2% v/v. The concentration in ppb (µg/L) of Y, Yb, Nd and Er was measured. Using the molecular formula of the matrix (1 mol Na : 1 mol Y : 4 mol F) and correcting by the dilution factor, the total mass in each sample was determined. Then, the mass of one nanoparticle (Eq. S1), the molecular weight of the NPs, the number of NPs per liter and the molarity were determined. For that, the NPs were considered spherical and the bulk densities for all the materials were applied: $\rho(NaYF_4)$ = 4.13 g/cm^3, $\rho(NaNdF_4)$ = 4.87 g/cm^3, ρ(Yb) = 6.98 g/cm^3, ρ(Er) = 9.05 g/cm^3, ρ(Er) = 7.01 g/cm^3.

$$m_{core} = V_{core} \cdot \rho_{core} \qquad \textbf{Eq. S1}$$

$$m_{core} = \left[\frac{4}{3}\pi\frac{d^2}{2}\right] \cdot [\rho(Yb) \cdot Yb_{\%} + \rho(Er) \cdot Er_{\%} + \rho(NaYF_4) \cdot Y_{\%}]$$

To determine both, core composition and concentration, cores from three independent synthesis were measured (Table S3). Using Eq. S1, the experimental percentage for each element (Table S4) and the measured diameter obtained by TEM (*d*, Table S5), the mass of an individual core (m_{NP}) and the molecular weight (MW) was determined. Results confirmed that the concentration of the core solutions dispersed in 30 mL of chloroform is 660± 44 nM (c_{NP}, Table S5).

	C_Y [10^3 µM]	C_{Na} [10^3 µM]	C_F [10^3 µM]	C_{Yb} [10^3 µM]	C_{Er} [10^3 µM]
1	(44.89 ± 0.37)	(44.89 ± 0.37)	(179.6 ± 1.5)	(5.11 ± 0.11)	(1.37 ± 0.02)
2	(46.35 ± 0.84)	(46.35 ± 0.84)	(185.4 ± 3.7)	(5.21 ± 0.12)	(1.51 ± 0.04)
3	(40.86 ± 0.37)	(40.86 ± 0.37)	(163.4 ± 1.5)	(4.58 ± 0.89)	(1.29 ± 0.02)

Table S3. Experimental concentrations of each element in three different syntheses of core UCNPs.

	Y [%]	Yb [%]	Er [%]	d [nm]
1	(87.38 ± 0.43)	(9.95 ± 0.03)	(2.67 ± 0.24)	(21.9 ± 1.7)
2	(87.34 ± 0.51)	(9.81 ± 0.21)	(2.85 ± 0.30)	(22.0 ± 1.3)
3	(87.45 ± 0.22)	(9.80 ± 0.07)	(2.75 ± 0.13)	(21.12 ± 0.89)

Table S4. Experimental concentrations of each element in three different synthesis of core UCNPs and diameters obtained from TEM micrographs.

$Y_{\%}$	$Yb_{\%}$	$Er_{\%}$	MW [$\cdot 10^{7}$ g/mol]	m_{NP} [$\cdot 10^{-17}$ g]	C_{NPs} [nM]
87.39 ± 0.06	9.85 ± 0.08	2.76 ± 0.09	(1.5 ± 0.1)	(2.32 ± 0.19)	660 ± 44

Table S5. Experimental values obtained by ICP-MS after performing statistics calculations.

In order to calculate the experimental element ratios in each shell, each synthetic step was analyzed independently. The expected UCNPs structure is $NaYF_4{:}Yb_{18\%}Er_{2\%}$@$NaYF_4{:}Yb_{10\%}$@$NaNdF_4{:}Yb_{10\%}$@$NaYF_4{:}Yb_{10\%}$. Using the experimental element percentage, the matrixes molecular formula and the Equations S2, the mass corresponding to each shell was determined ($m_{shell,x}$). These values, combined with the shell thickness and volumes, allowed for the total mass of the final core@shell$_1$@shell$_2$@shell$_3$ calculation (Eq. S2).

$$m_{UCNP} = m_{core} + m_{shell1} + m_{shell2} + m_{shell3} \qquad \textbf{Eq. S2}$$

$$m_{shell1} = \frac{\pi}{6}(d^3_{shell1} - d^3_{core}) \cdot [\rho(Yb) \cdot Yb^{\%}_{shell1} + \rho(NaYF_4) \cdot Y^{\%}_{shell1}]$$

$$m_{shell2} = \frac{\pi}{6}(d^3_{shell2} - d^3_{shell1}) \cdot [\rho(Yb) \cdot Yb^{\%}_{shell2} + \rho(NaNdF_4) \cdot Nd^{\%}_{shell2}]$$

$$m_{shell3} = \frac{\pi}{6}(d^3_{shell3} - d^3_{shell2}) \cdot [\rho(Yb) \cdot Yb^{\%}_{shell3} + \rho(NaYF_4) \cdot Y^{\%}_{shell3}]$$

	CORE	$SHELL_1$	$SHELL_2$	$SHELL_3$
**$Y_{\%}$**	(83.79 ± 0.01)	(94.20 ± 0.64)	-	(95.30 ± 1.60)
**$Yb_{\%}$**	(13.59 ± 0.01)	(5.80 ± 0.10)	(6.15 ± 0.20)	(4.67 ± 0.19)
**$Er_{\%}$**	(2.62 ± 0.01)	-	-	-
**$Nd_{\%}$**	-	-	(93.85 ± 0.19)	-
**Element Ratio**	Y/Er = 31.91 ± 0.05<br>Yb/Er = 5.17 ± 0.01	Y/Yb = 16.25 ± 0.28<br>Y_{shell1}/Y_{core} = 1.66 ± 0.02	Nd/Yb = 15.25 ± 0.50	
**Diameter [nm]**	24.0 ± 1.1	42.1 ± 6.5	52.3 ± 2.1	59.3 ± 9.4
**Mass [$\cdot 10^{-18}$ g]**	33.5 ± 4.8	137 ± 78	178 ± 100	147 ± 230

Table S6. Dopants percentages and dopants ratios within the shells measured by ICP-MS obtained for core, core@shell$_1$, core@shell$_1$@shell$_2$ and core@shell$_1$@shell$_2$@shell$_3$. Diameters of the samples as determined by TEM and the mass of an individual particle of each sample.

d [nm]	MW [$\cdot 10^7$ g/mol]	m_{NP} [10^{-16} g]	$m_{Er/NP}$ [10^{-18} g]	$m_{Nd/NP}$ [10^{-18} g]	C_{NPs} [nM]
59.3 ± 9.4	(30 ± 16)	(4.9 ± 2.6)	(1.71 ± 0.14)	(162.92 ± 0.56)	**160 ± 14**

Table S7. Characteristic values for the final core@shell$_1$@shell$_2$@shell$_3$ UCNPs.

	$M_{Er/NP}$ [10^{-17} g]	C_{NPs} [nM]
core	(0.128 ± 0.014)	**660 ± 44**
core@multishell	(0.128 ± 0.014)	**160 ± 14**

Table S8. NPs concentration for final samples of cores and core@shell$_1$@shell$_2$@shell$_3$ structures. Comparison of concentrations.

Absorbance measurements. A Biochrom Libra S60 UV-visible spectrophotometer was used to record UV/Vis absorption spectrum of the final solutions of cores and core@shell$_1$@shell$_2$@shell$_3$ (core@multishell) diluted in chloroform to a final concentration of 25 nM of NPs.

Photoluminescence measurements. The photoluminescent properties of the UCNPs, cores and core@multishell, were studied in a Horiba FluoroMax-3 spectrometer equipped with an optical fiber attached to a 980 nm or an 808 nm laser. When a laser was used as light source, the spectrometer lamp was disabled. 5 nM solutions of the samples were measured using a laser power of 1 W$\cdot$cm^{-2}.

IV. Synthesis and characterization of water dispersable UCNPs and surface modification

a) Polymer synthesis and previous modification of photosensitizers

<u>*Scheme of the synthetic process for the PS modification to produce azide-modified PS*</u>

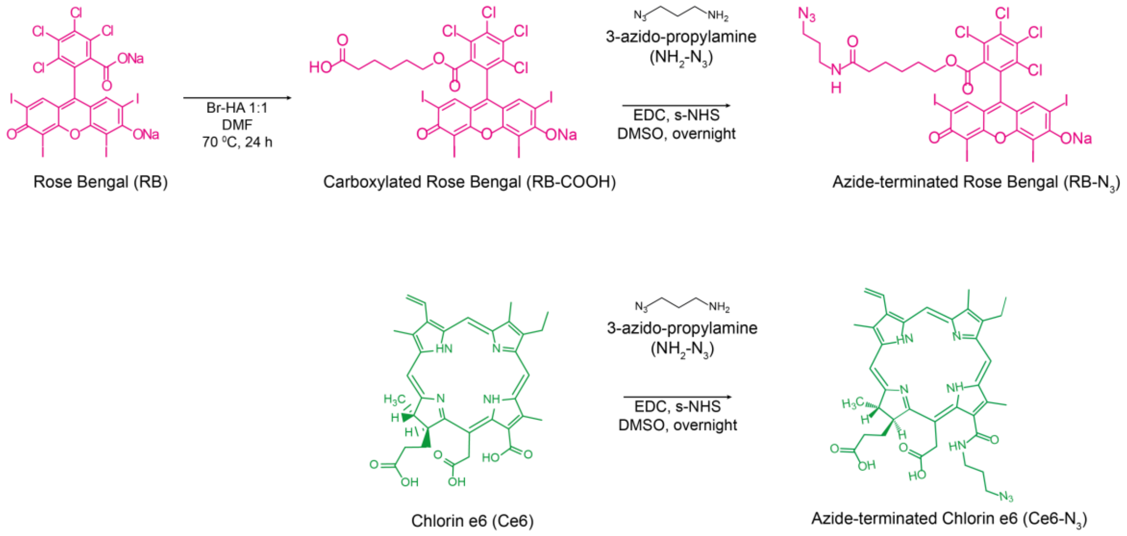

Figure S5. Scheme of the synthetic process for the PS modification to produce azide-modified PSs.

<u>*Absorbance spectrum of PS-N_3.*</u> A Biochrom Libra S60 UV-visible spectrophotometer was used to record UV/Vis absorption spectrum of the final solutions of the modified dyes. Stocks in DMSO were diluted in water to a final concentration of 20 µM (10% of DMSO) and placed in a quartz cuvette with 1 mL of sample.

<u>*DBCO-modified PMA preparation.*</u> An amphiphilic polymer was synthesized to further modify the UCNPs to make them colloidally stable and to provide them with reactive groups to carry out click chemistry reactions. Figure S6 depicts the synthetic process to produce DBCO-modified PMA.

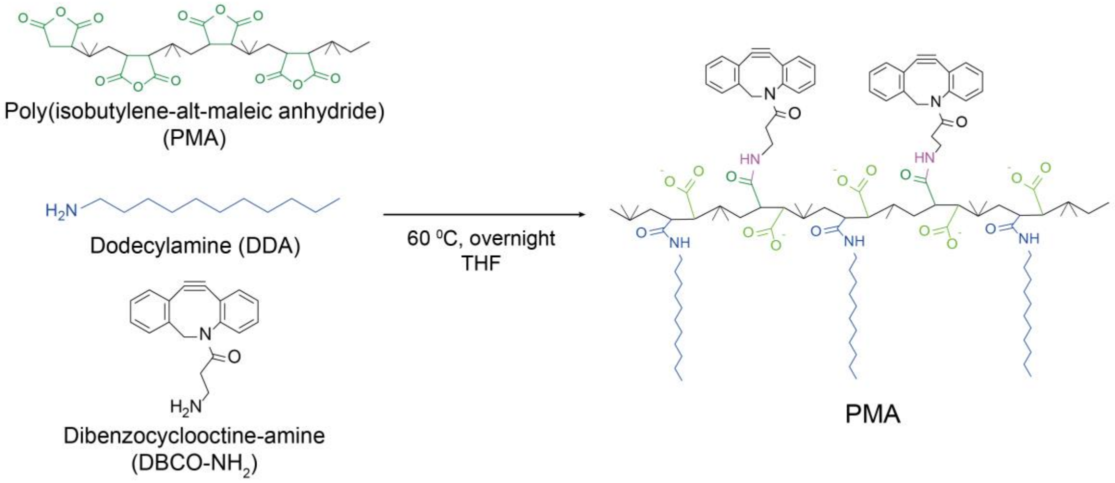

Figure S6. PMA preparation and modification for further NPs functionalization and click chemistry onto their surface.

b) PMA functionalization of oleic acid caped UCNPs

PMA coating of UCNPs. The UCNP diameter (*d*) (Table S6) was used to determine the amount of required PMA monomers per cm^2 (R). Thus, to calculate the amount of polymer (V_P) needed to stabilize a determine volume of a solution of UCNPs (VN_P) with a determine concentration (C_{NP}), the Equation S3 was used. Based on our previous work [1], an R of 500 monomers/cm^2 was selected. The effective diameter, d_{eff}, corresponds to the experimental diameter plus twice the length of the organic ligands (*ca.* 1.2 x 2 nm, *i.e.*, oleic acid).

$$V_P = \frac{R \cdot \pi \cdot d_{eff} \cdot C_{NP} \cdot V_{NP}}{C_P} \quad \textbf{Eq. S3}$$

.

Emission spectra. Fluorescence spectroscopy measurements were carried out, with an 808 nm laser as excitation source to characterize the UCNPs photoluminescence properties in water and using xenon lamp to quantify the PS attached to the UCNPs. Measures were done in a quartz cuvette with 1 mL of sample at 5 nM of NPs. Figure S7 shows the emission spectra of a 5 nM solution of UCNPs before and after water transference.

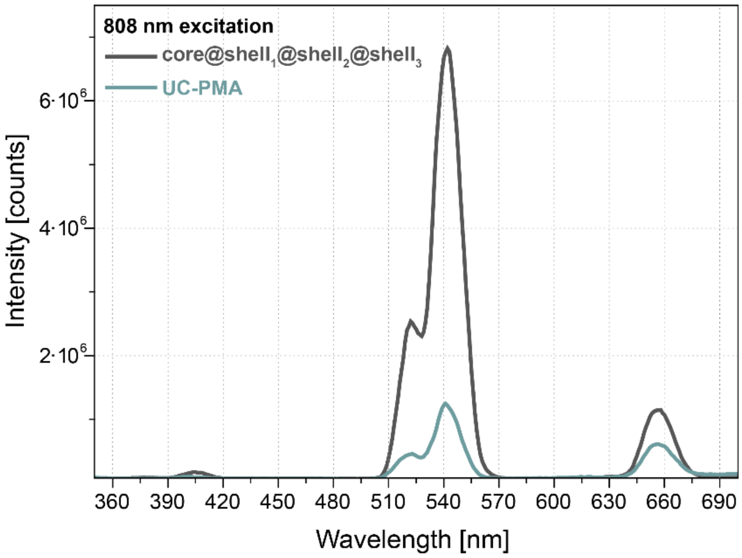

Figure S7. Fluorescence spectra of 5 nM solutions of UCNPs before (core@multishell) and after (UC-PMA) water transference.

c) Copper free click chemistry onto the UC-PMA

Conjugation of PS-N_3 and UC-PMA NPs. Figure S8 shows a photograph of the UC-PMA NPs conjugated with one or both PS.

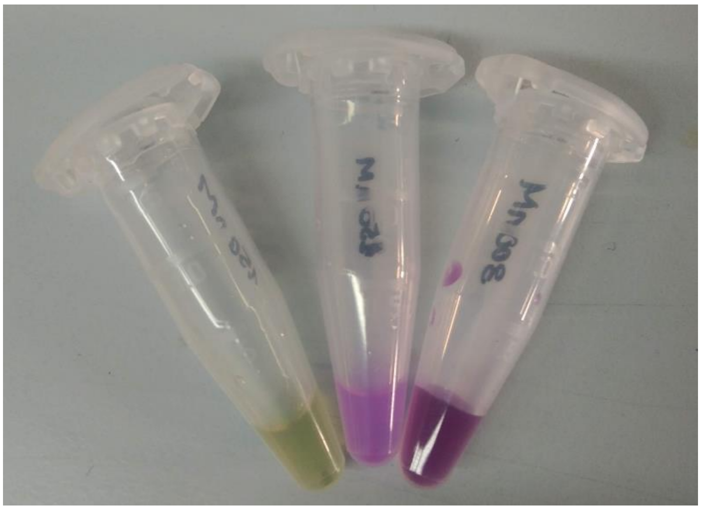

Figure S8. UC-PMA NPs conjugated with one PS type: from left to right, Chlorin e6 (UC-PMA-Ce6), Rose Bengal (UC-PMA-RB) or both PS. (UC-PMA-RB,Ce6)

Quantification of PS per UCNP. Calibration curves to determine the concentration of Rose Bengal and Chlorin e6 attached to the NPs were collected in water exciting at 540 nm and 400 nm, respectively. The emitted fluorescence at 567 nm and 658 nm for RB and Ce6, respectively versus the concentration was plotted (Figure S9). These curves were used to quantify the number of PS per UCNP.

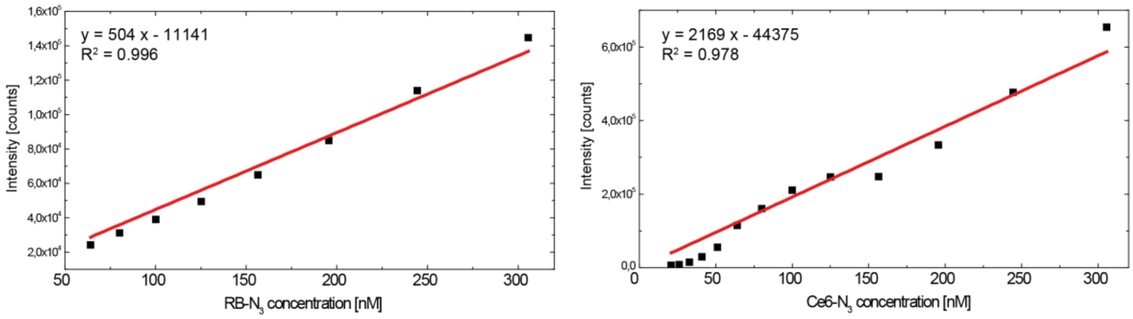

Figure S9. Calibration curves of RB (λ_{ex} = 540 nm, λ_{em} = 567 nm) and Ce6 (λ_{ex} = 400 nm, λ_{em} = 658 nm) in water.

d) Characterization of UCNP nanoplatforms

<u>*SEM.*</u> UC-PMA-RB,Ce6 NPs in water were observed under SEM to determine its size and monodispersity. Micrographs shown in Figure S10 confirm that morphology and colloidal monodispersity were kept after the water transfer and the bioconjugation of the PS.

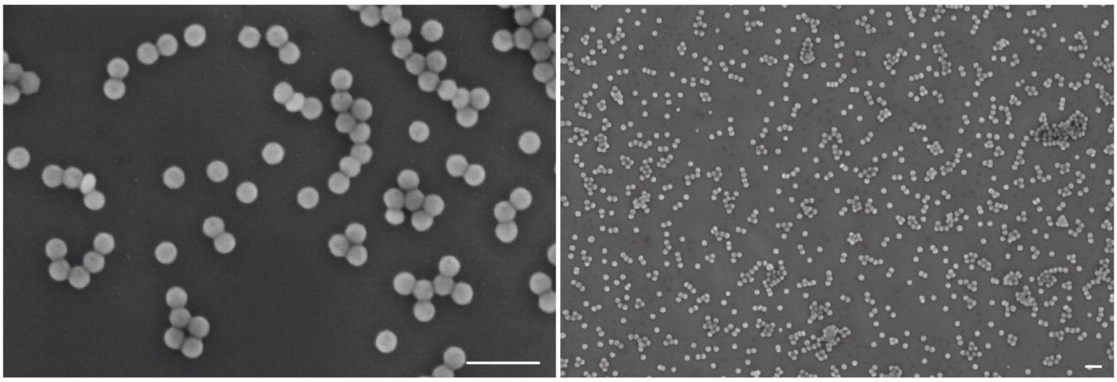

Figure S10. SEM micrographs of the final nanoplatform dispersed in water (UC-PMA-RB,Ce6) at different magnifications (scale bars represent 200 nm).

<u>*Dynamic light scattering and ξ-potential measurements*</u>. Hydrodynamic diameter and surface zeta potential (mean ± standard deviation) were measured in a Zetasizer Nano ZSP. Disposable cells for DLS and disposable capillary zeta cell for ξ-potential were used. 5 nM UCNPs solutions in water were used. Results are summarized in Figure S11.

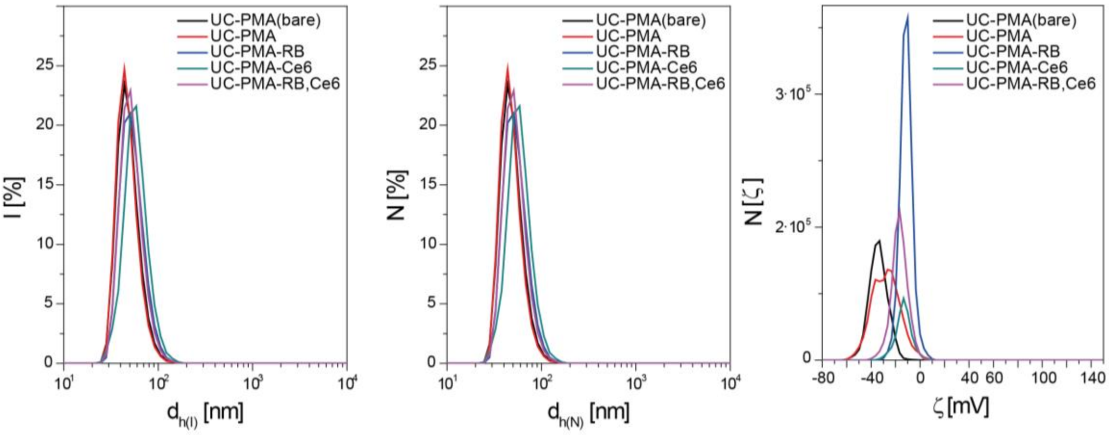

Figure S11. Intensity, Number and ξ-potential distributions for UC-PMA(bare), UC-PMA, UC-PMA-RB, UC-PMA-Ce6 and UC-PMA-RB,Ce6 NPs in MilliQ water.

The stability of the UCNPs in different biological relevant media was evaluated over time, up to seven days. Four different media were tested: i) water, ii) artificial lysosomal fluid (ALF), iii) ALF supplemented with a10% of FBS, iv) complete cell culture medium (Dulbecco's Modified Eagle Medium, DMEM, supplemented with 10% of FBS). Number mean values at each time are summarized in Table S9. Intensity and number distribution curves for the different media are summarized in Figure S12.

	**UC-PMA**			
	**water**	**ALF**	**ALF_{suppl}**	**$DMEM_{suppl}$**
**0 h**	62.90 ± 2.10	1614 ± 1580	65.80 ± 2.20	58.60 ± 14.40
**6 h**	48.54 ± 5.37	1348 ± 567	83.80 ± 5.70	60.88 ± 11.05
**24 h**	52.69 ± 4.70	728 ± 220	98.00 ± 6.65	60.80 ± 10.30
**48 h**	48.96 ± 3.50	188.5 ± 376.0	94.40 ± 16.80	59.70 ± 19.60
**72 h**	49.50 ± 1.60	139.6 ± 311.0	94.40 ± 12.90	59.65 ± 7.70
**7 days**	51.20 ± 4.30	0.00 ± 0.02	98.60 ± 8.20	57.45 ± 28.74
	**UC-PMA-RB,Ce6**			
	**water**	**ALF**	**ALF_{suppl}**	**$DMEM_{suppl}$**
**0 h**	54.80 ± 5.80	125.60 ± 15.98	100.10 ± 43.60	102.50 ± 46.24
**6 h**	58.30 ± 6.20	212.50 ± 54.45	108.50 ± 14.00	118.20 ± 30.90
**24 h**	58.80 ± 5.80	383.80 ± 129.70	115.30 ± 43.60	110.20 ± 37.00
**48 h**	59.20 ± 3.50	361.50 ± 61.90	117.00 ± 35.3p	131.50 ± 11.60
**72 h**	60.04 ± 4.80	471.20 ± 55.29	122.00 ± 43.60	131.00 ± 21.80
**7 days**	56.80 ± 5.00	0.70 ± 0.02	107.50 ± 13.64	124.90 ± 5.90

Table S9. Raw hydrodynamic diameter values (number weighted) overtime in different media (water, ALF and ALF and DMEM both supplemented with a 10% FBS) of UC-PMA and UC-PMA-RB,Ce6.

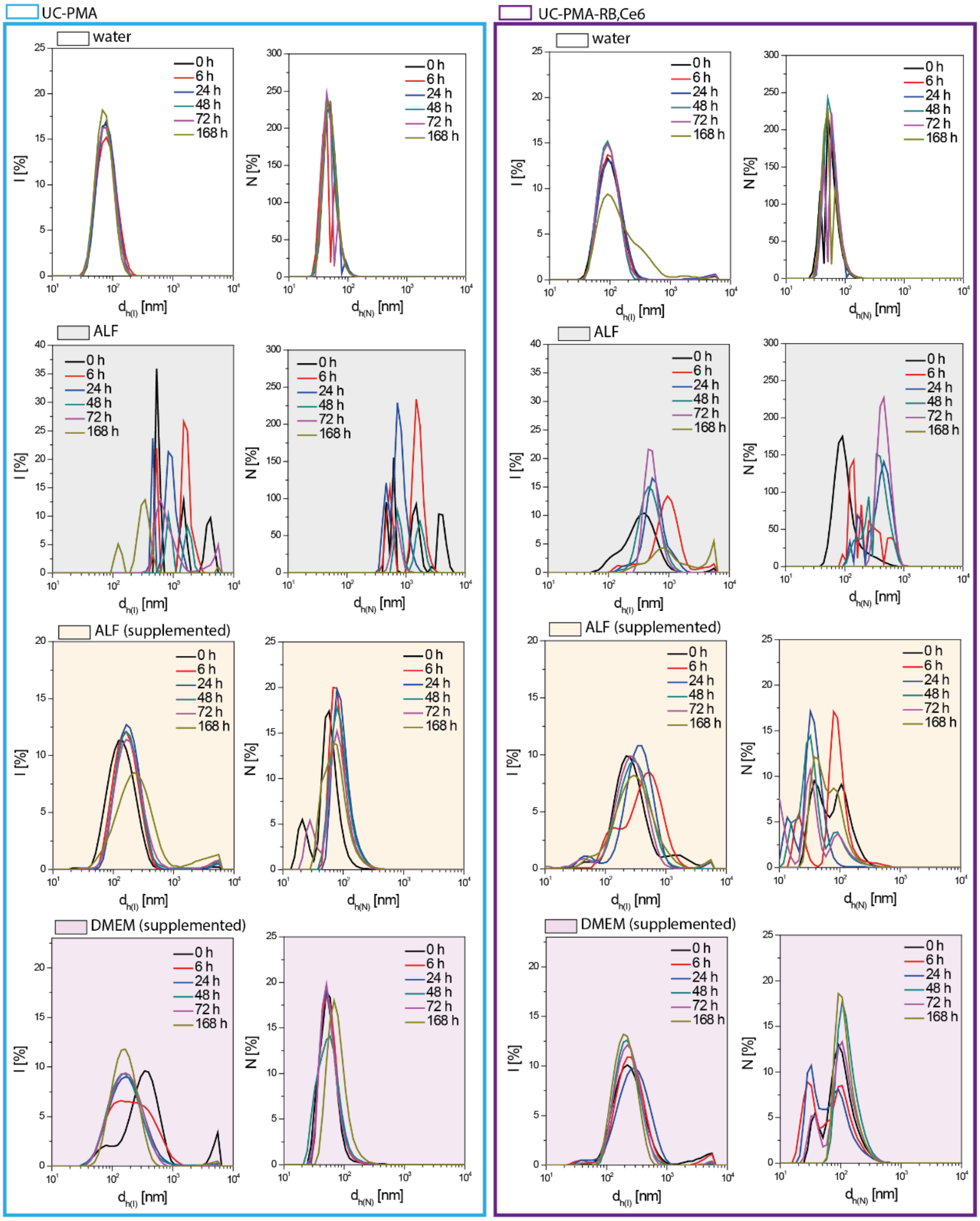

Figure S12. Raw intensity and number DLS values for UC-PMA and UC-PMA-RB,Ce6 during 7 days in different media (water, ALF, ALF supplemented and DMEM supplemented).

V. Photodynamic therapy activation by 808 nm irradiation and ROS production

<u>*Laser irradiation experiments:*</u> Figure S13 shows the homogeneous spot and its size produced by an 808 nm collimated laser used for cell irradiations.

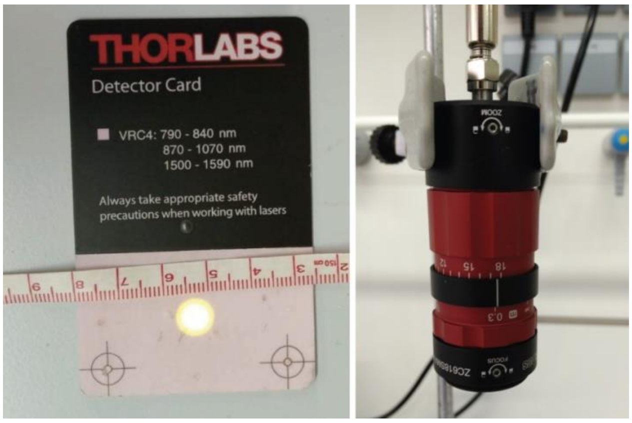

Figure S13. Image of spot diameter using a NIR detector card (left) and the collimation/zoom system (right).

<u>*Nanoparticles internalization studies.*</u>

ICP-MS studies. Table S10 summarized the total number of internalized NPs by cell as determined via ICP-MS.

	**Total NP/cell**
Control cells	0.44 ± 0.14
Cells + 5 nM of NPs	$(13.38 \pm 0.47)\cdot 10^3$

Table S10. Internalized UCNPs per cell determined by ICP-MS analysis.

Confocal imaging of living cells. In Figure S14 and S15, cells were incubated with UC-PMA-RB,Ce6 under strict dark conditions. Also, a different experiment was performed (Figure S16) using only UC-PMA-RB to avoid overlapping between channels.

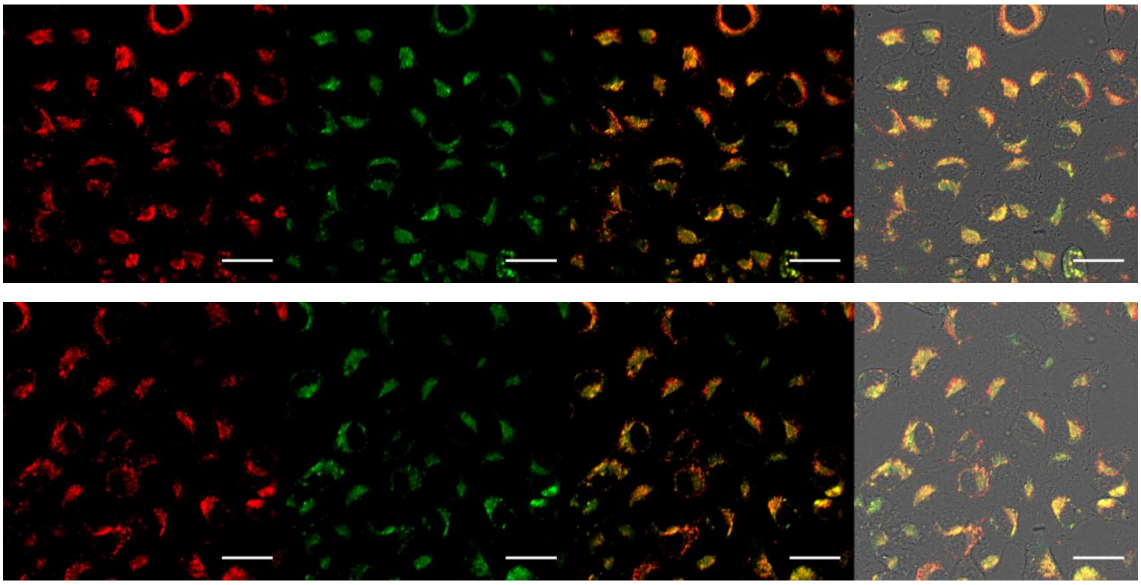

Figure S14. Intracellular location of UC-PMA-RB,Ce6 in HeLa cells after 3 h of incubation (2.5 nM of NPs). From left to right: Red (RB, Ex. 561, Em 620/60), Green (Ce6, Ex. 405, Em. 725/40), merged Red+Green (yellow color indicated colocalization of RB and Ce6), merged image of bright field (BF) + red + green. 60X magnification images (scale bars represent 40 µm).

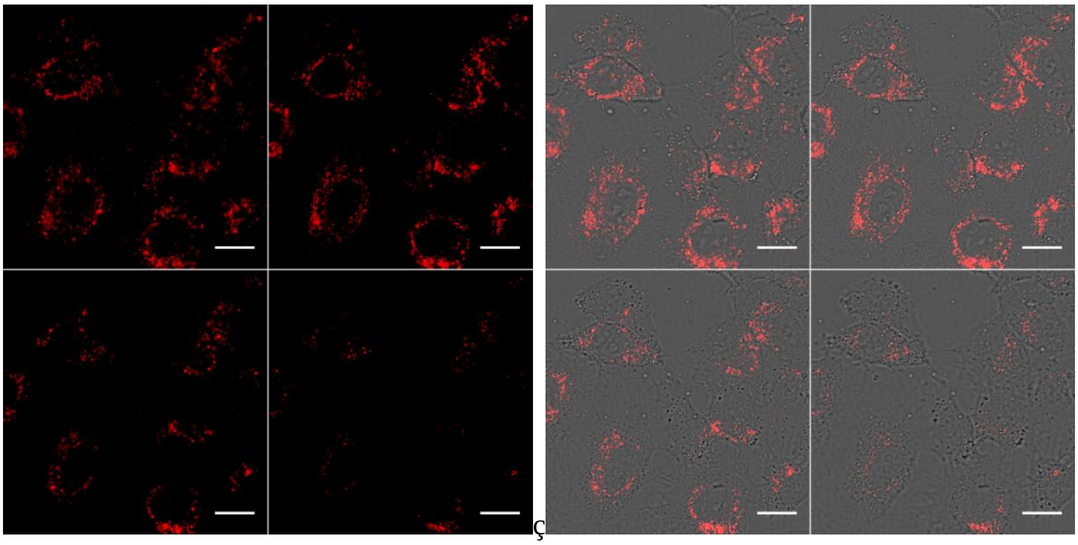

Figure S15. Intracellular location of UC-PMA-RB,Ce6 in HeLa cells (z-scan, 4 images) after 3 h of incubation (2.5 nM of NPs). Red (RB, Ex. 561, Em 620/60) and merged image of BF+Red. Scale bars represent 20 µm.

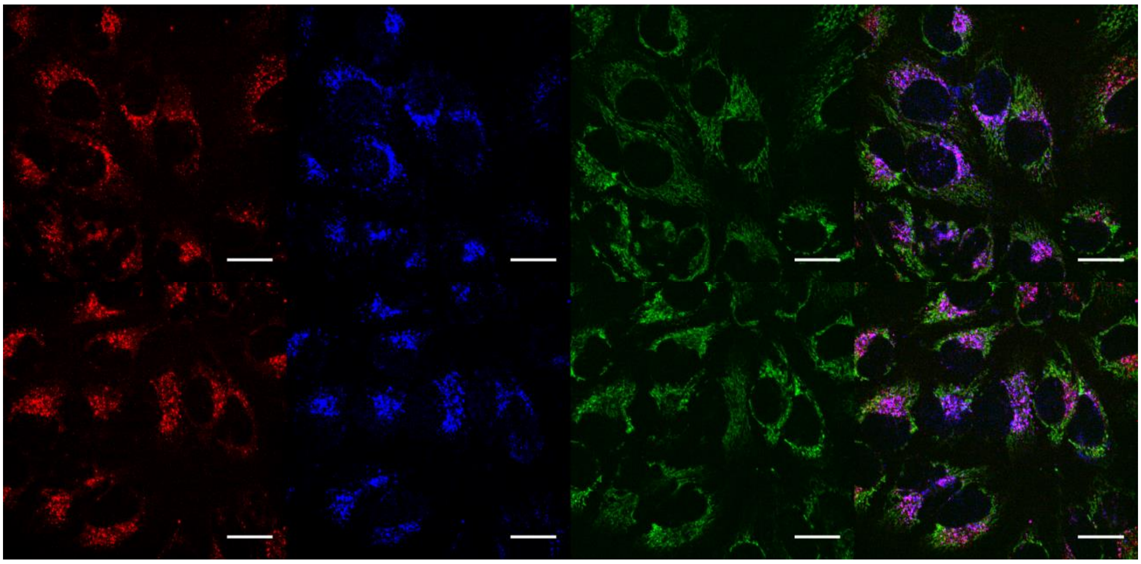

Figure S16. Intracellular colocation of UC-PMA-RB and lysosomes and mitochondria in HeLa cells after 3 h of incubation. From left to right: Red (RB, Ex. 561, Em 620/60), Blue (Lysotracker Blue, Ex. 405, Em. 450/50), Green (Mitotracker Green, Ex. 488, Em. 525/50), merged image of blue + red + green. Purple indicates lysosomes and UCNPs colocalization. Scale bars represent 40 µm.

ROS production measurements.

Figure S17 shows the variation of the absorbance spectra of DPBF in solution containing 2.5 nM UC-PMA-RB,Ce6 versus time upon 808-laser irradiation.

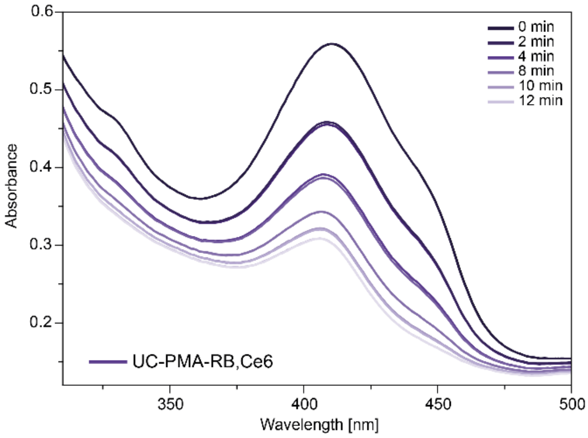

Figure S17. Degradation of DPBF due to the ROS production under 808 nm laser excitation of UC-PMA-RB,Ce6 NPs.

Confocal microscopy TMRE studies.

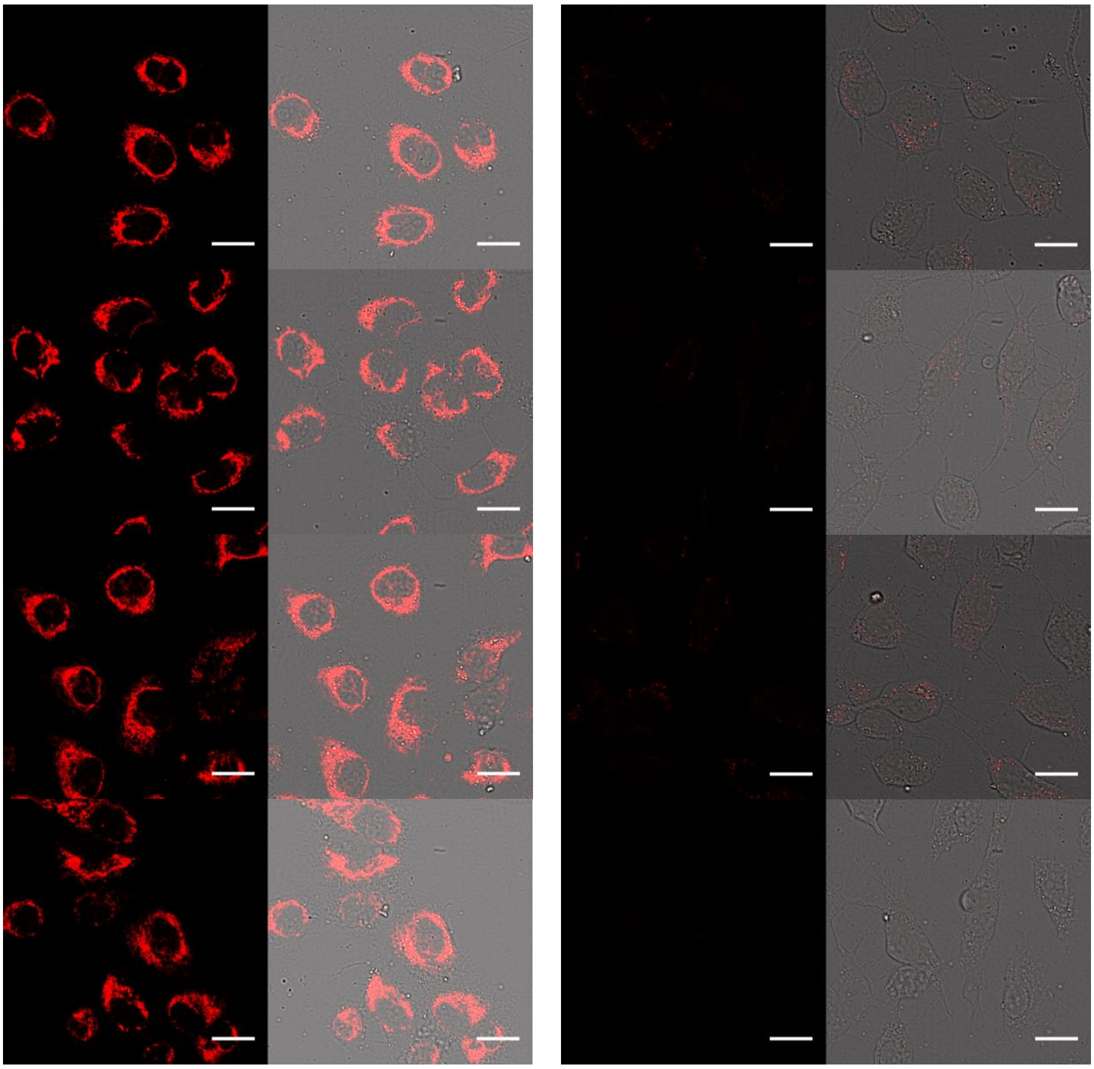

Figure S18. Cells treated with UC-PMA-Rb,Ce6; 2.5 nM during 3 hours under dark conditions after TMRE exposure (left). Cells treated in the same way but after 40 minutes of 5 W·cm^{-2} irradiation (right).
